# Oral Spermidine Supplementation Preserves Submandibular Gland Function After Radiotherapy: Mechanistic Insights and Use in a Phase II Randomized Clinical Trial

**DOI:** 10.1002/mco2.70862

**Published:** 2026-07-15

**Authors:** Yu Min, Kun Gao, Yingtong Liu, Lei Dai, Xingchen Peng

**Affiliations:** ^1^ Department of Biotherapy Cancer Center West China Hospital Sichuan University Chengdu China; ^2^ Sichuan Provincial Key Laboratory of Nuclear Physics and Medical Research Sichuan University Chengdu Sichuan China; ^3^ Department of Head and Neck Oncology Cancer Center West China Hospital Sichuan University Chengdu China; ^4^ Department of Biotherapy Cancer Center and State Key Laboratory of Biotherapy West China Hospital Sichuan University Chengdu China

**Keywords:** clinical trial, radiotherapy, spermidine, submandibular gland injury, xerostomia

## Abstract

Radiotherapy for head and neck cancer commonly induces salivary gland dysfunction, resulting in xerostomia that substantially impairs quality of life after treatment. To identify protective strategies, we established the irradiation‐induced submandibular glands (SMG) injury mice model and performed integrated proteomic and metabolomic profiling of gland tissue and saliva. Spermidine levels were significantly altered in irradiated SMG during the postradiotherapy recovery phase. Oral spermidine supplementation restored salivary flow, preserved acinar aquaporin‐5 expression, and attenuated vacuolization, apoptosis, and fibrosis in vivo; these effects were validated in SMG organoids. Single‐cell RNA transcriptomic analysis further showed that spermidine increased the proportion of acinar cells, enhanced Golgi‐mediated secretory gene expression, and suppressed inflammatory cytokine signaling. In the randomized, double‐blind, proof‐of‐concept clinical trial, oral spermidine supplementation during peri‐radiotherapy significantly reduced the incidence of xerostomia at 3 months compared with placebo (48.28 vs. 79.31%, *p* = 0.0391), while increasing salivary output and improving quality of life without serious adverse events. These findings highlight the potential of spermidine as a safe, metabolite‐based strategy for preserving salivary gland function and alleviating radiation‐induced xerostomia.

**Clinical trial registration**: The RCT has been registered in Clinical. gov (NCT07035626).

## Graphical abstract

Radiotherapy‐induced xerostomia severely impairs quality of life. Integrated multiomics, organoids, murine models, and a randomized Phase II trial identified spermidine as a key radiation‐responsive metabolite. Oral spermidine preserved salivary gland function, maintained acinar cell integrity through Golgi‐mediated posttranslational modifications, reduced inflammation, and safely alleviated xerostomia while improving salivary output in patients.

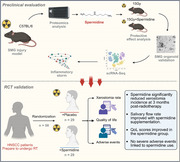

## 1 Introduction

Salivary glands play an important role in maintaining oral health [1, 2]. Adequate saliva secretion can not only facilitate food digestion and oral microbiome balance but also help with tooth cleaning and swallowing [1]. The major salivary glands, including the submandibular, sublingual, and parotid glands, regulate both unstimulated and stimulated saliva secretion [3]. Especially, the bilateral submandibular gland (SMG) is responsible for producing unstimulated saliva, making it essential for lubricating the oral cavity and facilitating swallowing [4]. Therefore, radiation‐induced dysfunction of the SMG, primarily caused by acinar cell injury, vascular damage, and progressive fibrosis, would lead to acute and chronic xerostomia in patients with head and neck cancer undergoing radiotherapy, thereby further impairing their quality of life (QoL) [1, 5, 6].

To date, radiotherapy is a cornerstone strategy in dealing with predominant head and neck cancers, offering curative potential either as a standalone modality or in combination with other therapies, while also serving as a palliative measure in advanced‐stage cancers [7, 8, 9, 10, 11, 12]. Although great progress has been achieved in radiotherapy techniques, injury to the salivary glands remains a prevalent complication among patients with head and neck cancer [13, 14, 15]. Due to the high sensitivity to radiation of salivary glands, acute‐phase cellular injury and vascular damage, as well as fibrosis, may contribute to substantial declines in salivary secretion [1, 4, 15]. Previous studies have demonstrated that nearly 50–60% of patients suffering from salivary flow rate decreases after head and neck radiation [16]. Consequently, it can induce irreversible SMG hypofunction and xerostomia. Emerging evidence has suggested that some treatment modalities, such as intensity‐modulated radiotherapy, stem cell transplantation, and pharmacological interventions (pilocarpine and cevimeline) would help to alleviate the radiotherapy‐induced SMG injury [16, 17, 18, 19]. Nevertheless, the effectiveness of these therapeutic modalities remained challenged and may not be the optimal strategy for SMG injury prevention or treatment [13, 18, 19, 20, 21].

Therefore, exploring the molecular mechanisms underlying radiotherapy‐induced SMG injury would be pivotal for identifying the key pathological processes and new therapeutic targets. With the advancements in omics techniques, researchers have ways to gain a deeper understanding of the mechanisms of various diseases [22, 23]. Integration of multiple technologies has emerged as an approach to provide a more comprehensive view of human health [22, 23]. Although recent studies have provided important insights into radiation‐induced salivary gland injury, the integrated molecular landscape of SMG damage and its translational validation remain insufficiently characterized [24, 25, 26]. In addition, most available studies have primarily emphasized specific cell populations or isolated mechanistic aspects, whereas an integrated framework linking molecular profiling with functional validation is still lacking [24, 25, 26]. Therefore, further investigation is warranted to bridge this gap and identify clinically relevant strategies for managing radiotherapy‐induced SMG damage. Moreover, clinical trials are essential for validating preclinical findings and providing stronger evidence of translational relevance, efficacy, safety, and feasibility of potential interventions [27, 28]. This integrated approach is expected to facilitate the development of more effective clinical strategies for radiation‐induced SMG injury and ultimately improve the QoL of patients with head and neck cancer undergoing radiotherapy.

In this study, we employ multiomics approaches to investigate the molecular mechanisms of radiotherapy‐induced SMG injury. Besides, we evaluate the therapeutic efficacy of identified spermidine on SMG organoids and in vivo models. More importantly, we also conduct a double‐blind, randomized clinical trial (RCT) to validate the clinical utility of spermidine for the prevention and treatment of radiotherapy‐induced SMG injury. This bench‐to‐bedside study would provide new insights into managing radiotherapy‐induced SMG injury and improve the QoL for the affected population.

## 2 Results

### 2.1 Dysregulated Arginine Metabolism Contributes to Radiation‐Induced SMG Injury

To investigate the radiation‐induced adaptive response in SMG, a mouse model was established. Specifically, the C57BL/6 male mice were exposed to localized radiation with a single dose of 15 Gy to induce SMG damage (Figure S1A). During the postirradiation days, salivary flow rates were measured. In the health control group, the average salivary flow rate was nearly 25 mg/min. By contrast, the salivary flow rate in the irradiated group significantly decreased to 18.5 mg/min on Day 1, 11 mg/min on Day 4, 10.5 mg/min on Day 8, and further declined to 9.3 mg/min by Day 22 without recovery (Figure S1B). The images of hematoxylin–eosin (H&E) staining showed that the damage to the SMG increased gradually after radiation, including the signs of acinar duct atrophy and acinar vacuolation (Figure S1C). Correspondingly, AQP5 expression decreased after irradiation, indicating impaired acinar integrity and secretory dysfunction in the SMG (Figure S1D,E). Meanwhile, α‐SMA expression increased after irradiation, suggesting aggravated stromal fibrotic remodeling in the SMG (Figure S2A,C). Moreover, the TUNEL staining suggested increased cell death in SMG after irradiation (Figure S1B,D). Therefore, we successfully established the radiation‐induced SMG injury model with significant functional impairment and histopathological changes.

**FIGURE 1:**
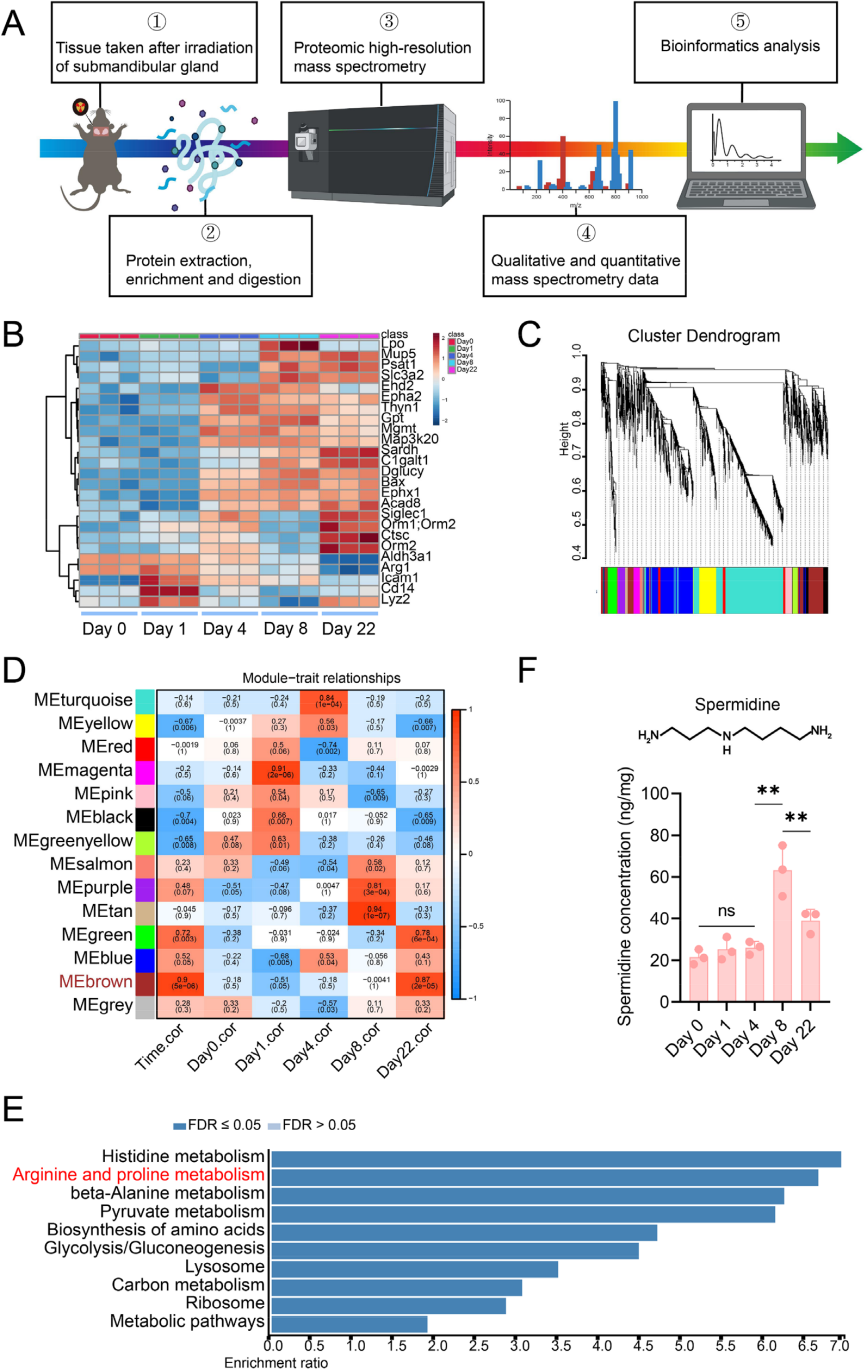
Identification of the dynamic changes in proteins during the SMG injury process. (A) Study protocol. (B) Heat map showing the dynamic changes of the top 25 differentially expressed proteins at different postirradiation days. (C) Weighted Correlation Network Analysis (WGCNA) to identify time‐related protein modules. (D) The coexpressed protein modules are at specific stages. (E) Metabolically related pathways involved in the SMG injury process. (F) Temporal changes in spermidine levels during the SMG injury process. One‐way analysis of variance (ANOVA) was used for group comparisons, followed by posthoc multiple‐comparison testing. Data are presented as mean ± SD. ns, not statistically significant; ^**^ *p* < 0.01.

To explore the underlying molecular mechanisms of SMG injury induced by radiation. SMG tissues were collected at 0, 1‐, 4‐, 8‐, and 22 days postirradiation and proteomic analysis was conducted to reveal the dynamic changes in proteins during the SMG injury process (Figure 1A). Among the top 25 differentially expressed proteins, aldehyde dehydrogenase 3A1 and arginase‐1 were highly expressed in the Day 0 group. Cluster of differentiation 14 was upregulated on Day 1. EH domain‐containing protein 2 was prominently expressed on Day 4. Lactoperoxidase was elevated on Day 8, and Orosomucoid 1 and 2 (Orm1, Orm2), along with cathepsin C, were upregulated on Day 22 (Figure 1B). These findings indicate that the protein expression profile of the murine SMG undergoes dynamic changes during the progression of radiation‐induced injury.

Furthermore, volcano plots were used to visualize differentially expressed proteins at each time point (|log2FC| > 1, *p* < 0.05), and bar plots were generated to illustrate the results of pathway enrichment analysis. Specifically, early‐stage injury (Day 1) was characterized by the upregulation of cholesterol metabolism‐related proteins. Mid‐stage injury (Day 4) showed activation of pathways related to N‐glycan biosynthesis, protein processing, and calcium reabsorption (Figure S3A,B). Late‐stage injury (Days 4–22) demonstrated enrichment of pathways involving antigen processing and presentation, as well as platelet activation (Figure S3C,D). Volcano plots and pathway enrichment analysis confirmed significant time‐dependent alterations in protein expression, reflecting the complexity and dynamic nature of protein networks in radiation‐induced SMG injury (Figure S3A–D).

To identify time‐dependent differential metabolites associated with radiation‐induced SMG injury, differential expression proteins were analyzed using the Weighted Correlation Network Analysis to identify time‐related gene modules (Figure 1C). Pathway enrichment analysis was further conducted to identify key pathways. Based on average hierarchical clustering and dynamic tree cutting, the consensus identifies 14 modules. As shown in Figure 1D, the brown module has the highest correlation with time (correlation = 0.9, *p *< 0.001), indicating that it may play a positive regulatory role in the process of radiation damage. Additionally, four metabolically related pathways, including histidine metabolism, arginine and proline metabolism, β‐alanine metabolism, and pyruvate metabolism, were identified (Figure 1E). Metabolomic profiling suggested that spermidine levels exhibited a time‐dependent pattern during radiation‐induced SMG injury (Figure S4). Subsequently, spermidine levels in SMG tissues were evaluated at different postirradiation time points, showing a transient increase at Day 8 followed by a gradual decline at later time points (Figure 1F). These findings suggest that spermidine may be involved in the temporal progression of radiation‐induced SMG injury.

### 2.2 Spermidine Supplement Alleviates Radiation‐Induced SMG Injury In Vivo and In Vitro

**FIGURE 2:**
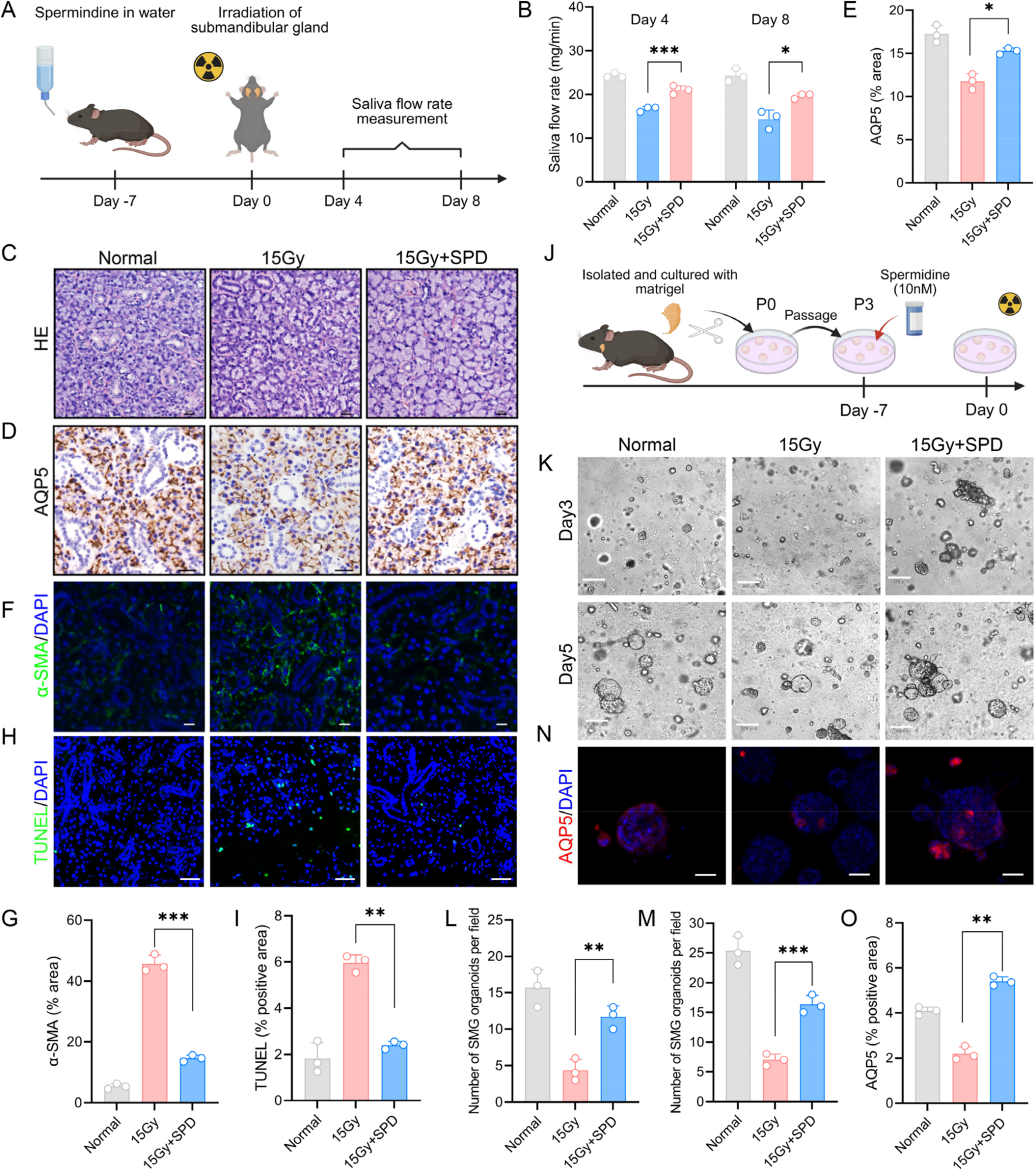
Protective effect of spermidine on radiation‐induced SMG injury. (A) Study protocol. (B) Salivary flow rate on Day 4 and Day 8 between different treated groups (*n* = 3). (C) Representative H&E images of acinar cells in different groups (*n* = 3). Scar bar = 50 µm. (D) Representative IHC images of AQP5 expression in different groups and (E) corresponding semi‐quantitative analysis (*n* = 3). Scar bar = 50 µm. (F) Representative immunofluorescence images of α‐SMA expression in different groups and (G) correspondingly semi‐quantitative analysis (*n* = 3). Scar bar = 50 µm. (H) Representative images of TUNEL expression in different groups and (I) corresponding semi‐quantitative analysis (*n* = 3). Scar bar = 100 µm. (J) The scheme of construction of SMG organoids and the spermidine treatment process. (K) Number of SMG organoids (scar bar = 200 µm) on postirradiation Day 3 and Day 5, and (L, M) corresponding semi‐quantitative analysis (*n* = 3). (N) Expression levels of AQP5 (scar bar = 10 µm) with (O) semi‐quantitative analysis (*n* = 3). One‐way analysis of variance (ANOVA) was used for group comparisons, followed by posthoc multiple‐comparison testing. Data are presented as mean ± SD. ^*^ *p* < 0.05; ^**^ *p* < 0.01; ^***^ *p* < 0.001.

To evaluate the protective effect of spermidine on radiation‐induced SMG injury, the mouse model with preventive spermidine treatment was set (Figure 2A). Mice received spermidine (3 mM) in drinking water starting 7 days before irradiation, with replenishment every 2 days. The salivary flow rate was assessed on Days 4 and 8 postirradiation, respectively. On Day 4, the average salivary flow rate was 16.6 mg/min in the irradiation‐only group, compared with 21.3 mg/min in the spermidine supplement group (*p* < 0.001) (Figure 2B). On Day 8, the average salivary flow rate was 15.0 mg/min in the irradiation‐only group and 19.6 mg/min in the spermidine supplement group, respectively (*p* = 0.021) (Figure 2B). These results preliminary suggested that oral spermidine supplements may help to prevent the onset of radiation‐induced xerostomia. Histologically, the acinar cells in the healthy control group exhibited intact morphology, with well‐developed ductal cells and visible zymogen granules (Figure 2C). In contrast, the irradiation‐only group displayed ductal dilation and vacuolation of acinar cells, whereas these pathological changes were markedly reduced in the spermidine‐supplemented group. Correspondingly, the positive area of AQP5 in the irradiation‐only group was 11%, while it increased to 14% in the spermidine‐supplemented group (*p* < 0.05; Figure 2D,E). The immunofluorescence staining revealed that the average α‐SMA‐positive area in the spermidine‐supplemented group was significantly lower than that in the irradiation‐only group (*p* < 0.001) (Figure 2F,G). More importantly, the percentage of TUNEL‐positive cells in the irradiated group alone was about 6.12%, while the spermidine supplementation group significantly decreased to 2.15% (*p* < 0.01) (Figure 2H,I). These results suggest that spermidine supplements during the peri‐radiotherapy period can help effectively preserve glandular architecture, protect acinar cells from radiation‐induced damage, mitigate radiation‐associated SMG fibrosis, and reduce cell apoptosis.

To further validate the protective effects of spermidine against radiation‐induced SMG injury, a spermidine preconditioning organoid model was established (Figure 2J). The results showed that by Day 3, the number of SMG organoids in the irradiation‐only group was lower than that in the spermidine‐treated group (Figure 2K,L). On day 5, the number of organoids in the irradiation‐only group was still lower than that in the spermidine‐treated group (Figure 2K,M). Furthermore, the mean AQP5‐positive area in SMG organoids was 3.1% in the irradiation‐only group, whereas it increased to 4.9% in the spermidine‐treated group (*p *< 0.01) (Figure 2N,O). These findings indicate that spermidine effectively restores the expression of AQP5 in the organoid model and prevents radiation‐induced SMG injury in vitro.

### 2.3 Spermidine Supplementation Restores Acinar Secretory Function by Activating Golgi‐Associated Pathways

**FIGURE 3:**
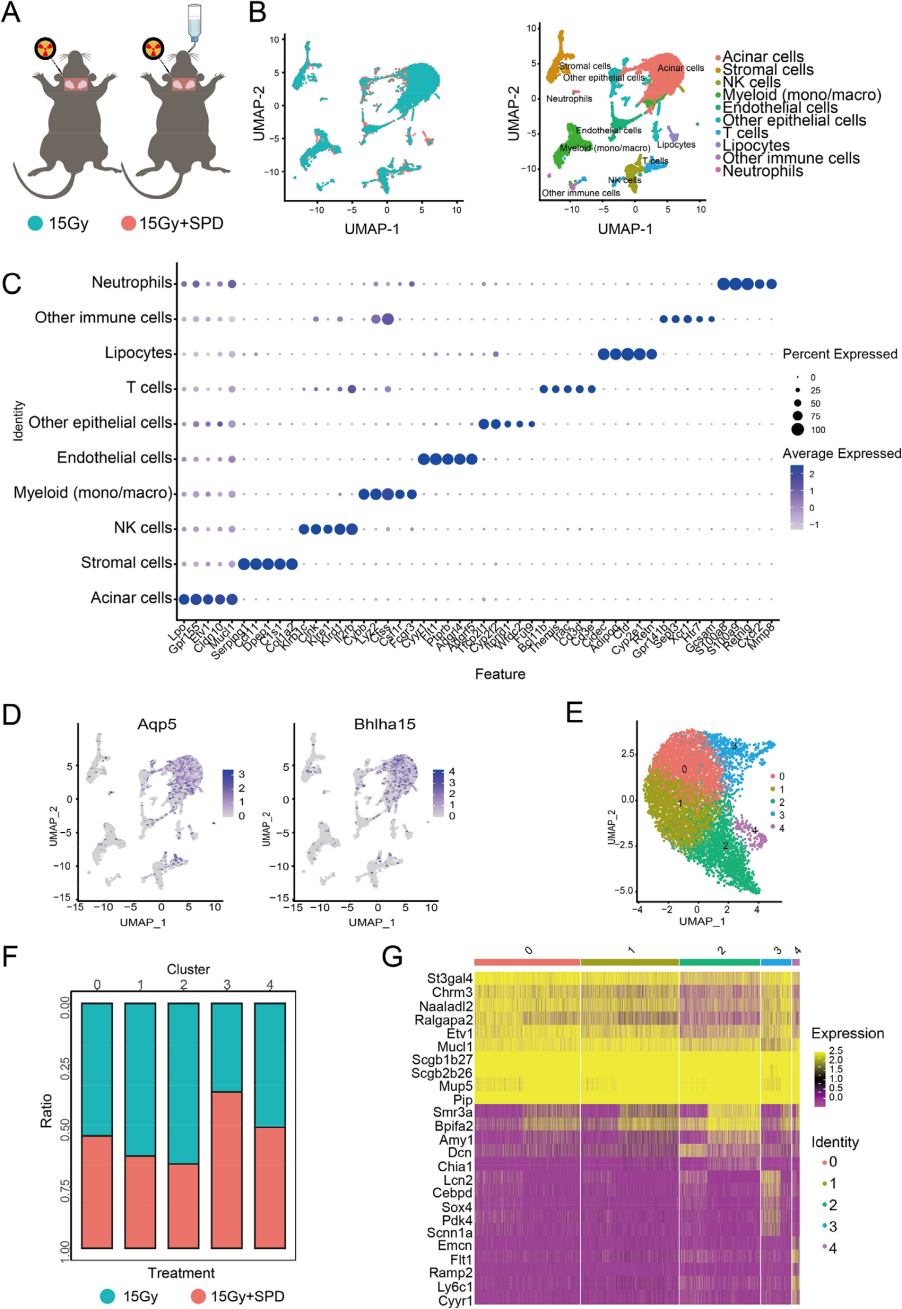
Single‐cell RNA (scRNA)‐seq reveals protective mechanism of spermidine against radiation‐induced submandibular gland injury. (A) Study protocol. (B) Distribution of subgroups of cells in 15 and 15 Gy + SPD groups. (C) Expression levels of cells in 15 and 15 Gy + SPD groups. (D) Distribution of acinar cell diversity. (E) Subgroups of the acinar cell population. (F) Clusters of different subgroups of cells in 15 and 15 Gy + SPD groups. (G) Heat maps showing the different expression levels of genes (yellow refers to up‐regulation and pink refers to downregulation).

To elucidate the protective mechanism of spermidine against radiation‐induced SMG injury, single‐cell transcriptome libraries were generated from SMG tissues collected from mice exposed to irradiation alone or pretreated with spermidine before irradiation (Figure 3A). After quality control, scRNA‐seq analysis identified 3303 cells in the spermidine‐pretreated group and 2458 cells in the 15 Gy irradiation‐only group. Dimensionality reduction and unsupervised clustering based on transcriptional similarity yielded distinct cell populations, including acinar cells (AQP5, BHLHA15), stromal cells (COL1A2, CCL11), natural killer cells (KLRD1, IL2RB), myeloid cells (CSF1R, LYZ2), endothelial cells (PTPRB, ADGRL4), other epithelial cells (Epcam, Sox9), lipocytes (CFD, ADIPOQ), T cells (CD3D, CD3E), neutrophils (S100A8/A9), and other immune cells (GPR141B, XCR1) (Figure 3B,C). Comparable cellular compositions were observed between the two groups, indicating that spermidine supplementation did not markedly alter the overall cell‐type distribution within SMG tissue.

To further investigate acinar cell heterogeneity, subgroup analysis was performed on the acinar population (Figure 3D,E). In the irradiation‐only group, acinar cells accounted for 49% of total cells, compared with 54% in the spermidine‐pretreated group. All acinar subpopulations expressed serious secretion markers such as prolactin‐induced protein (PIP) and carbonic anhydrase 6. Notably, clusters 0 and 1, which represent transitional states between ductal and acinar cells and are characterized by high Etv1 expression, accounted for 54 and 62% of cells in the spermidine group, respectively, but only 46 and 38% in the irradiation‐only group. Cluster 2, associated with salivary secretion and marked by high expression of amylase and SMG secretory protein, comprised 66% of cells in the spermidine group compared with 34% in the irradiation‐only group. In contrast, cluster 3, linked to innate immune responses and characterized by high lipocalin‐2 expression, was more prominent in the irradiation group (64%). Cluster 4, associated with angiogenesis and marked by FLT1 (VEGFR‐1) expression, accounted for 51% of cells in the spermidine group (Figure 3F,G). These findings suggest that spermidine pretreatment preserves acinar cell abundance and supports secretory function without substantially altering acinar subpopulation identity.

**FIGURE 4:**
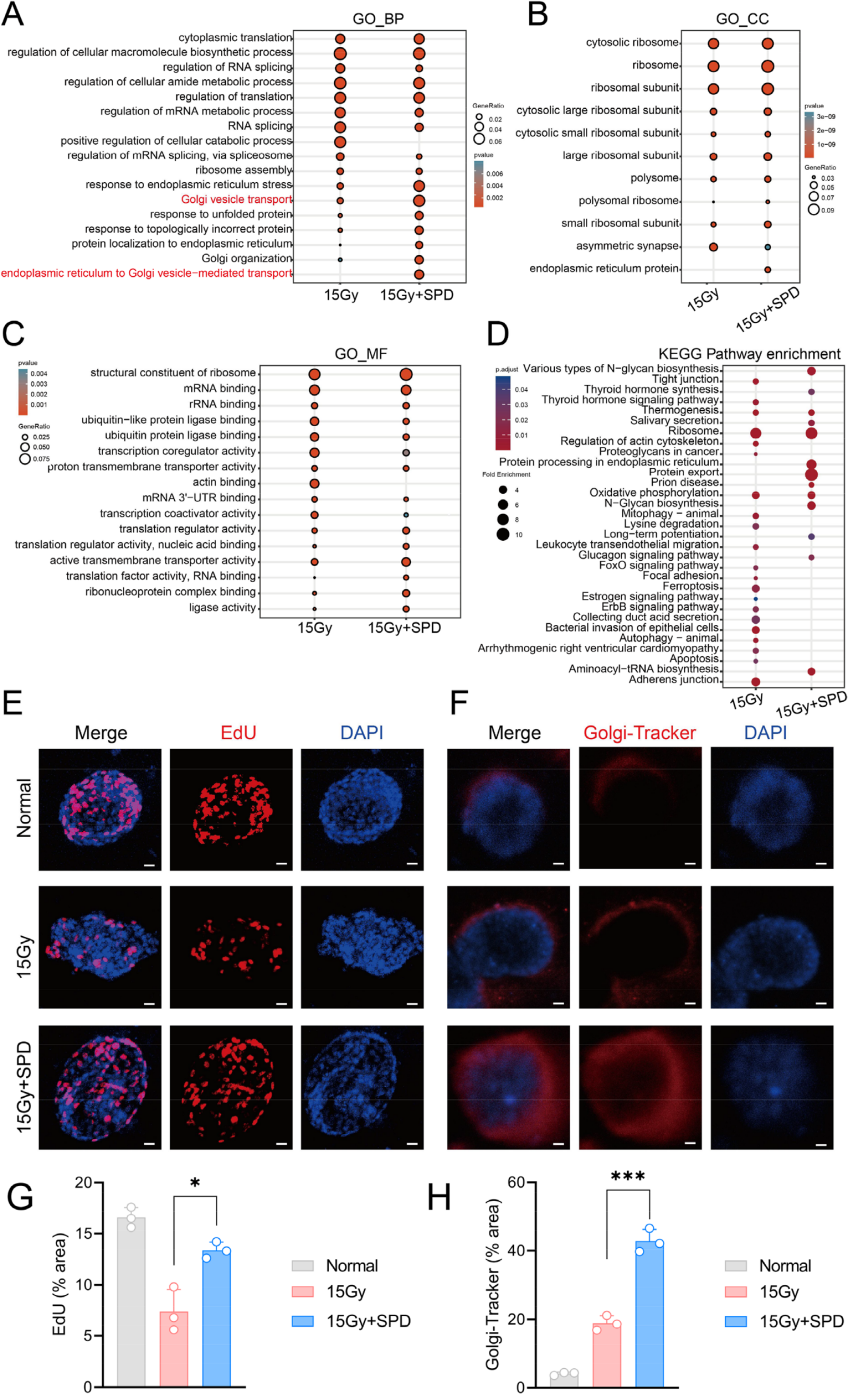
Enriched pathways involved in the protective mechanisms of spermidine in acinar cells. (A) GO (biological process) enrichment. (B) GO (cellular component) enrichment. (C) GO (molecular function) enrichment. (D) KEGG enrichment. (E) Representative immunofluorescence images of EdU staining in different groups and (G) corresponding semi‐quantitative analysis. Scar bar = 10 µm. (F) Representative immunofluorescence images of Golgi staining in different groups and (H) corresponding semi‐quantitative analysis. Scar bar = 10 µm. Student's *t*‐test was used for two‐group comparisons. Data are presented as mean ± SD. ^*^ *p* < 0.05; ^***^ *p* < 0.001.

To define the molecular programs underlying this protective effect, Gene Ontology (GO) and Kyoto Encyclopedia of Genes and Genomes (KEGG) analyses were subsequently performed on acinar cells. GO enrichment revealed upregulation of genes related to endoplasmic reticulum and Golgi vesicle transport in the spermidine group, whereas genes involved in macromolecular biosynthesis and catabolism were enriched in the irradiation‐only group (Figure 4A–C). KEGG analysis further showed that genes associated with N‐glycan biosynthesis and salivary secretion were preferentially activated in the spermidine group, while ribosomal and tight junction‐related pathways were enriched in the irradiation‐only group (Figure 4D). Together, these multiomics findings indicate that spermidine enhances glandular secretory capacity, potentially by promoting Golgi‐associated activity in acinar cells.

To validate this mechanism, Golgi staining and EdU‐based proliferation assays were performed in organoids. Golgi staining showed a sparse punctate distribution in nonirradiated organoids, increased Golgi aggregation after irradiation, and marked Golgi enrichment following spermidine supplementation. Although irradiation induced only a modest increase in Golgi presence, this change was insufficient to restore proliferative activity. In contrast, spermidine treatment led to pronounced Golgi enrichment and significantly restored organoid proliferation to near‐normal levels (Figure 4E–H). These results suggested that spermidine promotes acinar cell survival and functional recovery by enhancing Golgi‐associated secretory activity, thereby mitigating radiation‐induced SMG injury.

### 2.4 Spermidine Supplementation Reduced the Inflammatory Response in Irradiated SMG Tissue

**FIGURE 5:**
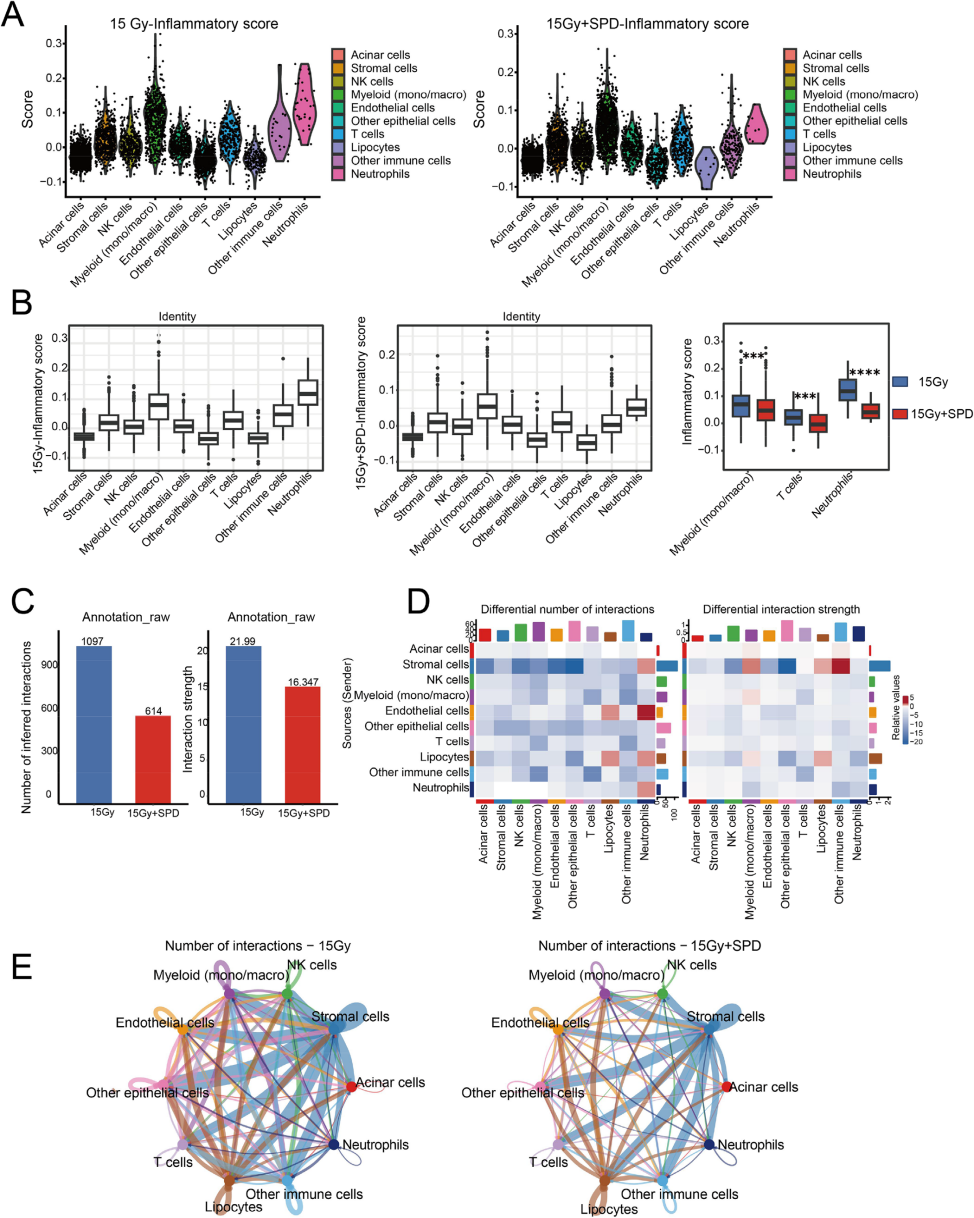
Enhanced inflammatory response in irradiated SMG and the potential role of spermidine. (A) Violin plot showing inflammation scores across major cell types in the 15 Gy and 15 Gy + SPD groups. (B) Box plot showing inflammation scores across major cell types with quantitative comparison. (C) Number of ligand–receptor pairs in the 15 Gy and 15 Gy + SPD groups. (D) Number of differential potential interactions between acinar cells and all other clusters. (E) Circular plot showing directional interactions among the identified cell types in the SMG. Data are presented as mean ± SD. ^***^ *p* < 0.001; ^****^ *p* < 0.0001.

Building on the transcriptomic findings, we next examined whether spermidine modulates the inflammatory microenvironment in irradiated SMG tissue. Inflammation scores were calculated for each cell type based on a predefined proinflammatory gene signature to assess their relative inflammatory activation. The results showed significantly elevated inflammatory cytokine gene expression in macrophages, neutrophils, and T cells, indicating that these populations were the major contributors to the heightened inflammatory response in irradiated SMG tissue (Figure 5A).

Importantly, prophylactic spermidine treatment markedly reduced the inflammation scores of these cell types, suggesting suppression of inflammatory activation response (Figure 5B). In addition, analysis of ligand–receptor interaction strength between cell populations revealed that intercellular communication was significantly attenuated in the spermidine‐treated group compared with the irradiation‐only group (Figure 5C–E). These data indicate that spermidine supplementation may alleviate irradiation‐induced inflammatory injury in SMG tissue by dampening inflammatory cell activation and weakening intercellular signaling within the inflammatory niche.

### 2.5 Oral Spermidine Supplementation Reduces the Incidence of Xerostomia

**FIGURE 6:**
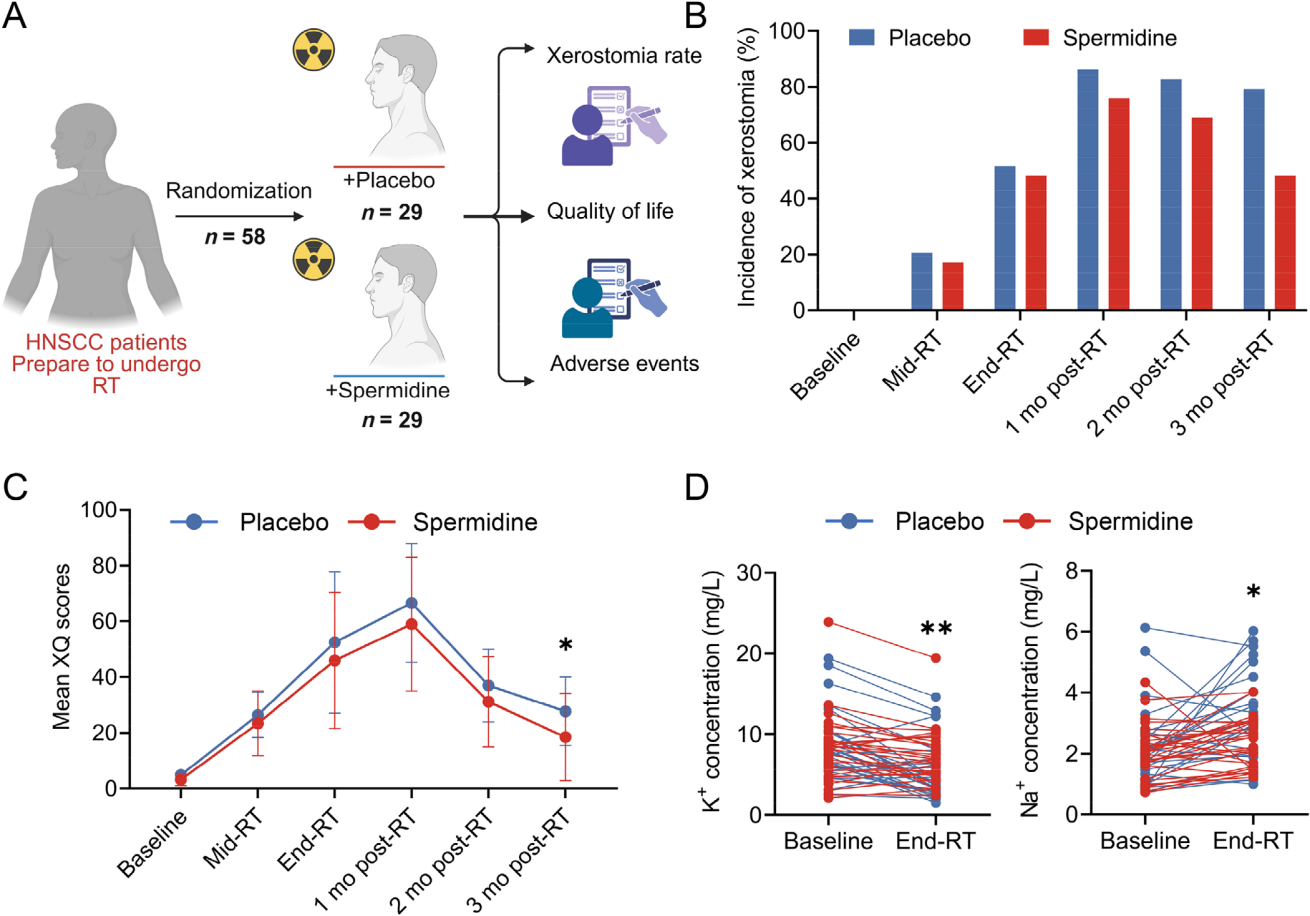
Protective effects of oral spermidine supplementation on xerostomia in patients with head and neck cancers undergoing radiotherapy. (A) Study design for evaluating the protective effects of oral spermidine supplementation on xerostomia. (B) Incidence of xerostomia determined by xerostomia questionnaire (XQ) scores at each time point in the placebo and spermidine groups (*n* = 29 in each group). (C) The specific XQ score of each participant in the placebo and spermidine groups (*n* = 29 in each group). (D) Longitudinal changes of salivary K^+^ and Na^+^ concentration (*n* = 29 in each group). Data are presented as mean ± SD. ^*^ *p* < 0.05; ^***^ *p* < 0.001. *p* Values are adjusted via the Benjamini–Hochberg method.

To further evaluate the clinical efficacy of oral spermidine supplementation in mitigating radiation‐induced SMG injury, we conducted a double‐blind randomized controlled trial (RCT) at one representative tertiary hospital in Southwest China. After eligibility screening, 58 patients scheduled to receive head and neck radiotherapy were enrolled and randomly assigned to either the placebo group or the spermidine supplementation group (Figures 6A and S5). Baseline characteristics were well balanced between the two groups and are summarized in Table 1. During radiotherapy and immediately upon treatment completion, no significant between‐group differences were observed in xerostomia incidence, xerostomia‐related symptoms, or salivary flow rate (Table S1). However, at 3 months after radiotherapy, the incidence of xerostomia was significantly lower in the spermidine group than in the placebo group (48.28 vs. 79.31%, *p* = 0.0391; Figure 6B and Table S1). Consistently, patients receiving spermidine reported significantly lower xerostomia questionnaire (XQ) scores, indicating milder subjective dry mouth symptoms (*p* < 0.05; Figure 6C). In addition, the unstimulated salivary flow rate was significantly higher in the spermidine group than in the placebo group at the 3‐month follow‐up (0.17 mL/min vs. 0.13 mL/min, *p*_adj_ = 0.0013; Table 2). These findings suggest that oral spermidine supplementation confers delayed but clinically meaningful protection against radiation‐induced xerostomia and promotes recovery of salivary gland function. Furthermore, salivary electrolyte analysis showed that, at 3 months after radiotherapy, patients in the spermidine group had significantly higher potassium (K^+^) levels and lower sodium (Na^+^) levels than those in the placebo group (Figure 6D), consistent with improved secretory composition and glandular function.

**TABLE 1:** Baseline patient characteristics.

	No. (%) of patients		
Characteristic	Spermidine (*n* = 29)	Placebo (*n* = 29)	All patients (*N* = 58)
Sex, no. (%)			
Male	22 (75.9)	23 (79.3)	45 (77.6)
Female	7 (24.1)	6 (20.7)	13 (22.4)
Age, years, mean (SD)	60.38 (10.83)	57.14 (13.16)	58.76 (12.06)
BMI, kg/m^2^, mean (SD)	22.04 (2.40)	22.30 (3.29)	22.17 (2.86)
ECOG PS, no. (%)			
0	26 (89.7)	26 (89.7)	52 (89.7)
1	3 (10.3)	3 (10.3)	6 (10.3)
Tumor pathologic types, no. (%)			
Squamous carcinoma	25 (86.2)	27 (93.1)	52 (89.7)
Others	4 (13.8)	2 (6.9)	6 (10.3)
Tumor site, no. (%)			
Nasopharynx	2 (6.9)	2 (6.9)	4 (6.9)
Oral cavity	12 (41.4)	11 (37.9)	23 (39.7)
Oropharynx	7 (24.1)	8 (27.6)	15 (25.9)
Hypopharynx	5 (17.2)	5 (17.2)	10 (17.2)
Larynx	3 (10.3)	3 (10.3)	6 (10.3)
Treatment type, no. (%)			
Definitive	5 (17.2)	6 (20.7)	11 (19.0)
Postoperative treatment	24 (82.8)	23 (79.3)	47 (81.0)
Concurrent chemotherapy, no. (%)			
Yes	7 (24.1)	6 (20.7)	13 (22.4)
No	22 (75.9)	23 (79.3)	45 (77.6)
TNM stage, no. (%)			
I–II	5 (17.2)	6 (20.7)	11 (19.0)
III	12 (41.4)	11 (37.9)	23 (39.7)
IV	12 (41.4)	12 (41.4)	24 (41.4)
Tumor primary site, no. (%)			
T1	2 (6.9)	3 (10.3)	5 (8.6)
T2	13 (44.8)	15 (51.7)	28 (48.3)
T3	10 (34.5)	9 (31.0)	19 (32.8)
T4	4 (13.8)	2 (6.9)	6 (10.3)
Nodal involvement, no. (%)			
N0	10 (34.5)	7 (24.1)	17 (29.3)
N1	9 (31.0)	10 (34.5)	19 (32.8)
N2	8 (27.6)	6 (20.7)	14 (24.1)
N3	2 (6.9)	6 (20.7)	8 (13.8)
Tobacco use, no. (%)			
Yes	15 (51.7)	16 (55.2)	31 (53.4)
No	14 (48.3)	13 (44.8)	27 (46.6)
Alcohol use, no. (%)			
Yes	11 (37.9)	11 (37.9)	22 (37.9)
No	18 (62.1)	18 (62.1)	36 (62.1)
Left parotid radiotherapy doses, cGy, median (IQR)	3449.00 (482.00)	3232 (563.00)	3393.50 (559.75)
Right parotid radiotherapy doses, cGy, median (IQR)	3182.00 (354.00)	3142 (475.00)	3158.00 (435.50)
Mean parotid doses, cGy, median (IQR)	3348.50 (367.00)	3229 (470.00)	3319.25 (405.88)
Total RT dose, cGy, median (IQR)	6450.00 (300.00)	6300 (302.00)	6360.0 (365.00)

*Note*: Body mass index (BMI) is calculated by taking the weight in kilograms and dividing it by the square of the height measured in meters.

Abbreviations: ECOG PS, Eastern Cooperative Oncology Group performance status; IQR, interquartile range; RT, radiotherapy; SD, standard deviation.

**TABLE 2:** Unstimulated salivary flow rate in two groups.

Time	Group	Sample size	USFR (mL/min, median (IQR))	95%CI	*p*	*p*_adj_
Baseline	Spermidine	29	0.36 (0.09)	(−0.03, 0.04)	0.7261	0.7261
	Placebo	29	0.37 (0.08)			
Mid‐RT	Spermidine	29	0.16 (0.03)	(−0.04, 1.30 × 10^−5^)	0.0515	0.0630
	Placebo	29	0.14 (0.04)			
End‐RT	Spermidine	29	0.13 (0.08)	(−0.04, −6.13 × 10^−5^)	0.0429	0.0630
	Placebo	29	0.09 (0.04)			
One‐month post‐RT	Spermidine	29	0.07 (0.06)	(−0.04, 3.54 × 10^−5^)	0.0525	0.0630
	Placebo	29	0.06 (0.03)			
Two months post‐RT	Spermidine	29	0.15 (0.09)	(−0.09, −0.03)	0.0004	**0.0013**
	Placebo	29	0.09 (0.02)			
Three months post‐RT	Spermidine	29	0.17 (0.08)	(−0.08, −0.02)	0.0004	**0.0013**
	Placebo	29	0.13 (0.03)			

Abbreviations: 95%CI, 95% confidence Interval; IQR, interquartile range; *p*_adj_, adjusted *p* value via Benjamini–Hochberg method; RT, radiotherapy; USFR, unstimulated salivary flow rate.

### 2.6 Oral Spermidine Supplementation Improves QoL and Shows a Favorable Safety Profile

The patient‐reported QoL, adverse events, and short‐term survival outcomes were further assessed. Compared with the placebo group, patients in the spermidine group demonstrated significantly better global health status (*p* < 0.05), less appetite loss (*p* < 0.05), improved head and neck swallowing function (HN‐swallowing, *p* < 0.01), and reduced dry mouth symptoms (HN‐dry mouth, *p* < 0.0001) at the end of the study (Figure S6A–L). Safety monitoring throughout the study showed no Grade 3 or 4 adverse events in either group (Table S2). Only a small number of patients in each group experienced mild abdominal discomfort, including distension and diarrhea. During the 3‐month postradiotherapy follow‐up, no recurrence or death was observed in either group (Figure S7A,B). Collectively, these findings support the favorable safety profile of oral spermidine supplementation and its potential to improve QoL in patients undergoing head and neck radiotherapy.

## 3 Discussion

Radiation‐induced SMG injury remains a major unmet clinical challenge in patients receiving head and neck radiotherapy. In this translational study, we established a representative in vivo model and, to our knowledge, performed the first integrated analysis of irradiated SMG tissue and saliva to define the molecular basis of salivary dysfunction. By combining omics profiling, organoid‐based validation, and a RCT, this work identifies the arginine–spermidine metabolic axis as a central regulator of radiation‐induced salivary gland injury and repair. These findings provide a preliminary mechanistic framework for understanding xerostomia and support oral spermidine supplementation as a biologically grounded and clinically feasible intervention.

Compared with previous saliva proteomics and integrative metabolomic–transcriptomic studies of parotid injury, which implicated glutathione depletion, mitochondrial dysfunction, and lipid metabolism alterations [29, 30], our data provide tissue‐intrinsic proteomic evidence across multiple functional pathways. By capturing proteomic shifts at 0, 1, 4, 8, and 22 days, the study reveals a coherent trajectory linking early disruption of arginine metabolism to radiation‐induced SMG injury. In particular, the early downregulation of arginine‐pathway enzymes complements our multiomics findings, highlighting spermidine dysregulation and suggesting coordinated metabolic‐proteomic perturbations underlying SMG dysfunction.

To date, spermidine has been recognized as a potent anti‐inflammatory mediator [31, 32, 33, 34]. An increasing number of preclinical works have shown that spermidine supplementation alleviates tissue damage and suppresses inflammatory responses [35, 36, 37], likely through enhanced mitochondrial metabolism [38], autophagy activation [39], and reduced radiation‐induced reactive oxygen species [33, 34, 40]. The association between spermidine and oral health has also begun to emerge. For example, Coeli‐Lacchini et al. reported that spermidine would help to prevent early oral carcinogenesis by inducing autophagy and reducing oxidative stress and DNA damage [41]. In lipopolysaccharide‐stimulated macrophages and microglial cells, spermidine suppresses NO and PGE2 production and downregulates IL‐6 and TNF‐α by blocking NF‐κB nuclear translocation as well as PI3K/Akt and MAPK signaling pathways. Notably, in marine models subjected to ionizing radiation, spermidine supplementation preserved thymic cellularity and naïve T‐cell pools while significantly dampening radiation‐induced proinflammatory gene expression in the thymus [42].

Furthermore, the radiation‐induced SMG injury models suggested that oral spermidine supplementation helps preserve AQP5 expression and maintain salivary flow during the peri‐radiotherapy period. Long‐term outcomes further showed reduced periductal fibrosis and acinar cell apoptosis, indicating improved glandular preservation. scRNA‐seq analysis additionally revealed increased acinar cell survival in the spermidine‐treated group, together with upregulated expression of Golgi‐associated posttranslational modification and secretion‐related genes. As a key organelle, the Golgi apparatus is responsible for terminal glycosylation and trafficking of surface proteins, such as growth factor receptors, which are essential for maintaining cellular homeostasis [43, 44]. Golgi dysfunction can therefore impair receptor activity, weaken signal transduction, and promote apoptosis [45, 46]. Previous studies have implicated the Golgi apparatus in membrane repair and cell proliferation [47], and Golgi‐related proteins such as CaMKK2, GOLIM4, and B4GALT1 have been shown to be indispensable for proliferation [48, 49]. Despite the limited temporal sampling and inherent constraints of the mouse model, these convergent preclinical findings consistently support a potential role for spermidine metabolism in radiation‐induced SMG injury, while also suggesting that its protective effects may involve coordinated modulation of acinar secretory programs and the inflammatory microenvironment.

Notably, the reduced ligand–receptor interaction strength observed in the spermidine‐pretreated group compared with the irradiation‐only group may reflect attenuation of excessive radiation‐induced inflammatory crosstalk, which is consistent with the concomitant reduction in inflammation scores across major immune populations. Similar reductions in immune‐cell‐centered intercellular communication networks have been associated with alleviated inflammatory responses and restoration of tissue homeostasis in other injured tissues [50, 51]. However, this finding should be interpreted cautiously, as immune‐cell interactions within the salivary gland are functionally heterogeneous. Especially, McKendrick et al. demonstrated that macrophage‐dependent signaling is indispensable for epithelial regeneration and functional recovery after irradiation, indicating that certain macrophage‐associated communication programs may be protective [26]. Therefore, the reduced communication inferred in the spermidine group may preferentially represent suppression of pathological inflammatory signaling rather than global inhibition of all macrophage‐mediated repair pathways. Given the likely coexistence of protective and deleterious immune‐cell subsets, future studies integrating subtype‐resolved spatial transcriptomics and functional perturbation will be required to define the precise communication networks that mediate injury versus regeneration in irradiated SMG tissue.

Given the well‐documented safety profile of spermidine as a dietary supplement and its anti‐inflammatory effects through the induction of functional autophagy [34, 39, 40], we conducted a single‐center, double‐blind, RCT to validate its protective effect against radiation‐induced SMG injury in a clinical setting. We conducted a single‐center, double‐blind RCT to validate its protective effect against radiation‐induced SMG injury in a clinical setting. Compared with the placebo group, patients receiving spermidine supplementation showed significantly lower XQ scores and only a mild decline in unstimulated salivary flow rate during radiotherapy. Biochemical analysis of saliva further revealed a favorable electrolyte profile in the spermidine group, with elevated K^+^ levels and reduced Na^+^ concentrations. Patients receiving spermidine also reported improved QoL, particularly in oral symptom‐related domains. At study end, no significant differences in short‐term tumor recurrence or overall survival were observed between groups, suggesting that spermidine supplementation does not compromise oncological safety while preserving salivary function. Collectively, these clinical findings support spermidine as a safe and clinically scalable adjunct with potential for routine integration into radiotherapy supportive care, pending validation in larger multicenter studies.

In summary, this study integrates multiomics analysis, in vitro and in vivo validation, and clinical investigation to reveal the protective role of spermidine supplementation in radiation‐induced SMG injury. Notably, to our knowledge, this is the first study to use an integrated multiomics approach to identify spermidine as a potential regulatory metabolite in radiation‐induced SMG injury. It is also the first study, to our knowledge, to employ murine SMG‐derived organoids to recapitulate key features of the salivary gland microenvironment and demonstrate that spermidine supplementation preserves SMG function under irradiation. Furthermore, through a single‐center clinical trial, we confirmed that oral spermidine supplementation reduces the incidence of radiation‐induced xerostomia and improves QoL for patients with head and neck cancer.

Several limitations should be acknowledged. First, although the multiomics analyses provided initial mechanistic insight, the precise molecular targets and downstream pathways of spermidine require further investigation. Second, only male mice were included in the preclinical experiments, and potential sex‐dependent differences in both radiation‐induced SMG injury and the therapeutic response to spermidine warrant future investigation. In addition, this was a single‐center‐based RCT with a relatively small sample size, which may limit the generalizability of findings. Although short‐term safety and efficacy were demonstrated, the long‐term outcomes and optimal dosing regimen of oral spermidine supplementation remain to be defined. For these reasons, future multicenter studies with larger cohorts and extended follow‐up are needed to validate our findings and further clarify the clinical utility of spermidine in mitigating radiotherapy‐induced oral gland toxicity.

## 4 Materials and Methods

### 4.1 Radiation‐Induced SMG Injury in the Mouse Model

C57BL/6 male mice aged 6–8 weeks with a body weight of approximately 20 g were purchased from Chengdu Dossy Experimental Animal Co., Ltd. The mice were kept under conditions of 25 ± 2°C with a humidity of 45–60%, with a 12‐h light/dark cycle, and had free access to food and water.

The mice were randomly divided into two groups, with six mice per group. All mice were anesthetized with 1% sodium pentobarbital. Except for the Day 0 group, all other groups received a single dose of 15 Gy irradiation. During irradiation, the mice were positioned supine and immobilized in a lead box. The upper lead shielding was adjusted to expose the SMG region. Irradiation was delivered at a dose rate of 1.14 Gy/min using an X‐ray generator (160 kV, 25 mA, 0.3 mm copper filtration). The setup is illustrated in Figure S1A. The salivary flow rate was used to measure the salivary secretion status, and fixation, embedding, sectioning, and immunofluorescence staining were used to evaluate the SMG tissue condition. All the reagents, antibodies, suppliers, and catalog numbers were listed in Tables S3 and S4.

### 4.2 Salivary Flow Rate Measurement

Salivary flow rate was measured at 0, 1, 4, 8, and 22 days, and a baseline measurement was obtained for each mouse before irradiation. Mice were fasted for 2 h before saliva collection and anesthetized by intraperitoneal injection of 1% pentobarbital sodium (100 µL). Salivary secretion was stimulated by intraperitoneal injection of 25 µL pilocarpine (0.5 mg/mL). Two minutes after pilocarpine administration, preweighed cotton was gently inserted into the oral cavity and left in place for 10 min to absorb saliva. The cotton was immediately reweighed after collection, and salivary flow rate (µL/min) was calculated as the difference between wet and dry cotton weights (mg) divided by the collection time (min), assuming a saliva density of 1 mg/mL.

### 4.3 Tissue Paraffin Embedding and Section

SMGs from different groups were collected from each mouse and fixed in 4% paraformaldehyde for 4 h. Tissues were washed with distilled water for 8 h, dehydrated with graded ethanol (75% overnight, 85% 1 h, 95% twice in 20 min, 100% three times in 5 min), cleaned with xylene (twice in 10 min), infiltrated with paraffin wax (40 min, 1 h, and 2 h, respectively), embedded, and sliced with a thickness of 4 µm. Slices are transferred to glass slides, heated in water at 39–42°C, loaded, and baked at 68°C for 1 h.

### 4.4 H&E Staining

The slices were roasted at 68°C for 4 h, then decarbonized in xylene (twice for 20 min twice) and passed through graded ethanol (100% twice, 5 min each time; 95% twice, 5 min each time; 85% and 75%, 5 min each). After 7 min of rinsing with distilled water, the sections were stained with hematoxylin for 5 min, washed with water, stained with eosin for 2 s, and sealed with neutral resin.

### 4.5 IHC Staining

After baking, deparaffinization, and rehydration, the sections were subjected to antigen retrieval by boiling in EDTA buffer (pH 9.0) for 5 min using a pressure cooker. Following retrieval, the sections were cooled to room temperature and washed with PBS three times for 3 min each. Blocking was performed with goat serum for 15 min at room temperature. Subsequently, 50 µL of primary antibody was applied to each section, and the sections were incubated overnight at 4°C. The next day, the sections were brought to room temperature for 30 min, washed with PBS, and incubated with enzyme‐conjugated secondary antibodies at 37°C for 1 h. After additional PBS washes, the DAB substrate was applied for chromogenic development. The sections were counterstained with hematoxylin, rinsed, air‐dried, and mounted with neutral resin. AQP5 expression was assessed by IHC as an indicator of acinar cell integrity and salivary secretory function.

### 4.6 Immunofluorescence Staining

After antigen retrieval, sections were blocked with goat serum for 1 h. Primary antibodies diluted in PBS were applied (50 µL per section) and incubated overnight at 4°C. Then, sections were washed, incubated with fluorescent secondary antibodies for 1 h at room temperature, and washed again in PBS. Sections were sealed with an antifluorescence quenching reagent containing DAPI and imaged. Immunofluorescence staining for α‐SMA and AQP5 was performed to evaluate stromal fibrotic remodeling and acinar cell integrity in the SMG, respectively. For α‐SMA quantification, only spindle‐shaped positive signals located in the interacinar stromal regions were analyzed, while vascular smooth muscle and perivascular structures were excluded to avoid confounding by physiological α‐SMA expression.

### 4.7 TUNEL Staining

TUNEL staining was performed to assess apoptosis levels. Organoids were first fixed with 4% paraformaldehyde at room temperature, followed by permeabilization with 0.2% Triton X‐100. After washing, the organoids were incubated with a TUNEL reaction mixture according to the manufacturer's protocol. Nuclei were counterstained with DAPI, and samples were mounted using an antifading agent. Images were captured under a fluorescence microscope, and apoptotic cells were identified by green fluorescence. Quantitative analysis of apoptotic cells was conducted using ImageJ software.

### 4.8 Proteomics and Metabolomics Sequencing

Ant Technology (Shanghai, China) conducted proteomics and metabolomics analyses. SMG tissues from each time point (0, 1, 4, 8, and 22 days postirradiation) were harvested and shipped on dry ice. For proteomics, proteins were extracted, digested, and analyzed using data‐independent acquisition. Metabolites were extracted and analyzed using data‐dependent acquisition for metabolomics. Raw data underwent preprocessing, including spectral peak processing and quality control, to identify high‐confidence metabolites for downstream bioinformatics analysis.

### 4.9 Concentrations of Spermidine in SMG Tissues

Spermidine levels in SMG tissues were quantified at different postirradiation time points (0, 1, 4, 8, and 22 days) and were quantified by high‐performance liquid chromatography–mass spectrometry system as described in previous literature [52]. Three biological replicates were analyzed per time point. The final spermidine concentrations were normalized to tissue weight (ng/mg).

### 4.10 Spermidine Pretreated Mice Model

To investigate the protective effects of spermidine, mice were divided into a spermidine‐supplemented group and a radiation‐only group. Mice in the spermidine group received 3 mM spermidine in drinking water for 7 consecutive days, with water replenished every 2 days to maintain stability and efficacy. This dose was selected based on prior preclinical studies of oral spermidine supplementation and was intended to model a practical preventive administration route [53, 54]. Mice in the control group received sterile water. Following preconditioning, all mice were exposed to radiation, and salivary flow rates were measured on Days 4 and 8 postirradiation to evaluate the salivary flow rate.

### 4.11 SMG Organoids

The SMGs were harvested and thoroughly rinsed with Hanks’ balanced salt solution (HBSS), and the tissues were minced into small fragments with scissors. The tissue fragments were digested in 2 mL digestion solution per gram of tissue at 37°C for 30 min on a shaker. The digestion solution consisted of HBSS supplemented with 1% bovine serum albumin (BSA), collagenase II (0.63 mg/mL), hyaluronidase (0.5 mg/mL), and calcium chloride (50 mM). The digest was filtered through a 100 µm cell strainer, and the filtrate was centrifuged at 350×*g* for 5 min to collect the cell pellet. The pellet was resuspended in 2 mL red blood cell lysis buffer, gently pipetted, incubated on ice for 2 min, and centrifuged again at 350×*g* for 5 min. The final cell suspension was adjusted to 2 × 10^5^ cells/mL. Then, 25 µL of the cell suspension was mixed with 50 µL of Matrigel on ice and seeded into the center of each well of a 48‐well plate. After gelation at 37°C for 30 min, organoid culture medium was added. Organoids were maintained at 37°C in 5% CO_2_, and the medium was changed every 3 days. For passaging, organoids were collected after 7 days of culture, dissociated into small clusters/single cells, and re‐embedded in fresh Matrigel under the same culture conditions. Organoids that had undergone three rounds of passaging and exhibited stable growth capacity were defined as third‐passage organoids (P3) and used for subsequent experiments. The organoid culture medium consisted of DMEM/F12 supplemented with 1% penicillin/streptomycin, 1× GlutaMAX, 50 ng/mL epidermal growth factor, 50 ng/mL fibroblast growth factor 2, 1× N‐2 supplement, 15 µg/mL insulin, 2 µM dexamethasone, 100 ng/mL hepatocyte growth factor, and 1 µM γ‐secretase inhibitor.

### 4.12 Spermidine‐Pretreated SMG Organoids

The P3 SMG organoids were pretreated with 10 nM spermidine. The irradiation‐only group served as the control. Spermidine treatment was initiated 7 days before irradiation and was replenished with each medium change to maintain a constant concentration.

### 4.13 Whole Amount of SMG Organoids Staining

Whole‐mount staining was employed to assess radiation‐induced damage in SMG organoids. The organoids were fixed with a solution of PIPES, MgCl_2_, EDTA, and paraformaldehyde, followed by washing and permeabilization with Triton‐based solutions. Blocking was performed using PBS with 5% BSA, and the organoids were incubated overnight at 4°C with the primary antibody. After washing and subsequent incubation with the secondary antibody at 37°C, the SMG organoids were mounted on concave slides with an antifade reagent for imaging.

### 4.14 ScRNA‐seq Analysis

Data quality control retained cells with 200–5000 detected genes (nFeature RNA), 5000–30,000 transcripts (nCount RNA), and <20% mitochondrial gene content. Normalization was performed using SCTransform, selecting 3000 highly variable genes for downstream analysis. Data integration was conducted with PrepSCT Integration, Find Integration Anchors, and Integrate Data. Dimensionality reduction and clustering were achieved through Run PCA and Run UMAP, with the first 30 dimensions used for clustering via Find Neighbors and Find Clusters, followed by UMAP visualization. Cell subpopulations were annotated based on marker genes identified by Find All Markers and COSG, providing robust subpopulation characterization.

Differential gene expression analysis utilized COSG, with pathway enrichment conducted through cluster Profiler for KEGG pathways. To evaluate inflammation‐associated cell types, the “HALLMARK INFLAMMATORY RESPONSE” gene set from MSigDB was used, and inflammatory scores were calculated with Seurat's Add Module Score. Highly inflammatory cell types were identified based on a median score above zero. For cell–cell communication, Cell Chat was used to analyze secreted signaling pathways using precompiled mouse protein–protein interaction networks. Key interactions and differences were identified with “compute Commun Prob Pathway,” “compute Commun Prob,” and “aggregate Net,” with differential interactions visualized through “compare Interactions” and “netVisual_diffInteraction.”

To evaluate inflammatory activity in irradiated SMG tissues, a single‐cell inflammation score was calculated using Seurat's AddModuleScore function based on a predefined proinflammatory gene signature, following the module‐scoring strategy described by Tirosh et al. and implemented in Seurat [55]. The score was computed as the average expression of the inflammatory gene set, corrected by matched control gene sets according to the standard Seurat pipeline. Cell‐type‐level inflammation scores were then obtained by averaging the scores of all cells within each annotated cell population. Higher scores indicate stronger proinflammatory activation. Differences in inflammation scores between groups were compared across the major immune cell populations, including macrophages, T cells, and neutrophils.

### 4.15 Golgi and Edu Staining

SMG organoids were stained using a red fluorescent Golgi apparatus probe and an EdU proliferation assay kit according to the study protocol provided by Beyotime (Biotechnology Co., LTD). Stained SMG organoids were counterstained with DAPI and mounted with the antifade reagent. Imaging was performed using fluorescence microscopy to assess Golgi apparatus distribution and cellular proliferation within organoids. All the reagents, antibodies, suppliers and catalog number used in this study were summarized in Tables S3 and S4.

### 4.16 Design and Results of a RCT

This was a double‐blind, placebo‐controlled superiority RCT. A total of 58 eligible patients from West China Hospital were enrolled and randomized to receive either spermidine or a placebo. Patients in the spermidine group were instructed to take spermidine capsules twice daily (total dose, 1 mg/day) with liquids from 1 week before radiotherapy until 1 week after radiotherapy completion, whereas the placebo group received identical placebo capsules on the same schedule. The primary endpoint was the mean XQ score, and the secondary endpoint was unstimulated salivary flow rate. Clinical xerostomia incidence was prespecified as the proportion of patients with an XQ score >30, representing clinically xerostomia. Exploratory endpoints included salivary electrolyte concentrations and QoL. Xerostomia was assessed weekly using the XQ scale during radiotherapy and for 3 months after radiotherapy. QoL was evaluated using the European Organization for Research and Treatment of Cancer (EORTC) Quality‐of‐Life Questionnaire Core 30 (EORTC QLQ‐C30) and Head and Neck Cancer Module (EORTC QLQ‐H&N35) at baseline and 3 months after radiotherapy. Reporting of the RCT followed the CONSORT guidelines [56].

All patients were randomized in a 1:1 ratio using a computer‐generated permuted block randomization list. Pharmacists not involved in trial implementation prepackaged spermidine and placebo in identical containers, which were distributed according to the randomization sequence. The two types of capsules were identical in appearance, taste, and color. The allocation list, block size, and random numbers were sealed in opaque envelopes and stored at West China Hospital, Sichuan University. Both participants and investigators were blinded to treatment allocation.

### 4.17 Sample Size Estimation

Sample size estimation was based on a superiority design. Assuming a xerostomia incidence of 75% in the placebo group and 50% in the spermidine group, with a minimum clinically relevant reduction of 10% in the spermidine group, PASS software (version 15.0) indicated that 24 participants per group were required to achieve 80% power at a two‐sided alpha level of 0.05 under a 1:1 allocation ratio. To account for an anticipated dropout rate of 20%, the target enrollment was set at 29 participants per group (total *n* = 58). All participants were included in the efficacy analyses according to the intention‐to‐treat principle. Longitudinal changes in questionnaire scores and salivary electrolytes across follow‐up time points were analyzed using a mixed‐effects model with fixed effects for treatment group, time, and group × time interaction, and a random effect for subject to account for within‐subject correlation. The model estimated predicted group means, mean differences, and corresponding *p* values.

### 4.18 Saliva Collection and Electrolyte Concentrations Analysis

Unstimulated whole saliva samples were collected via the spitting method at baseline and at 3 months postradiotherapy. All samples were immediately frozen at −20°C and transferred to −80°C for storage until further analysis. Saliva samples for salivary electrolyte analyses were obtained from all participants. All submitted samples were used for subsequent biochemical analyses.

### 4.19 Statistical Analysis

The preclinical results were statistically analyzed by GraphPad Prism software (version 8.0), and the difference between the two groups was compared by the Student‐*t* test. One‐way ANOVA analysis, following posthoc multiple‐comparison testing, was used to compare the differences among multiple groups. For clinical data, continuous variables were analyzed using the *t*‐test (parametric) or Wilcoxon rank‐sum test (nonparametric), while categorical variables were compared with *χ* ^2^ or Fisher's exact tests. The risk difference (RD) in xerostomia incidence between groups was evaluated with a prespecified superiority margin of 10%. Superiority was declared if the lower limit of the 95% confidence interval (CI) for RD exceeded 10%, indicating that the experimental group was statistically superior. This was supplemented by a one‐sided marginal Z‐test to assess statistical significance. The exploration analyses were not corrected for multiplicity and should be interpreted conservatively as evidence against the null hypothesis. Statistical analyses were performed using R software (version 4.4.0; R Foundation for Statistical Computing, Vienna, Austria).

## Author Contributions

Y.M., K.G., and Y.T.L. contributed equally to this work. X.C.P. and L.D. conceived the idea and designed the project. Y.T.L. and Y.M. performed the experiments and analyzed the results. K.G. and Y.M. designed and conducted the clinical trial. X.C.P. and L.D. assisted with the figure production and experimental design. All authors wrote the manuscript. L.D. and X.C.P. corrected the manuscript and supervised the whole project. All authors discussed the results and commented on the manuscript. Correspondence and requests for materials should be addressed to Prof. S.Y.Z. or X.C.P. All authors have read and approved the final manuscript

## Funding

The work was supported by the Noncommunicable Chronic Diseases‐National Science and Technology Major Project (2023ZD0503000 and 2023ZD0503004), the Regional Innovation and Development Joint Fund Key Project of the National Natural Science Foundation of China (U24A20735), the National Natural Sciences Foundation of China (82473434), Sichuan Science and Technology Program (2025YFHZ0087, 2024YFHZ0041, 2024ZYD0054), 1.3.5 project for disciplines of excellence from West China Hospital of Sichuan University (ZYYC23006), and Postdoctor Research Fund of West China Hospital, Sichuan University (2024HXBH141), The funders had no role in study design, data collection and analysis, decision to publish, or preparation of the manuscript.

## Ethics Statement

The research protocol was initially approved by the Ethics Committee of West China Hospital, Sichuan University (No. 2023–2055). Written informed consent was obtained from all participants. All the animal experiments were approved by the Ethics Committee of West China Hospital, Sichuan University (No. 20240612005).

## Consent

Informed consent was obtained from all individual participants included in the study.

## Conflicts of Interest

The authors declare no conflicts of interest.

## Supporting information




**Supplementary Fig. S1**: Construction of the SMG injury model in vivo. (A) Irradiation area for construction the SMG injury model in mice. (B) Salivary flow rates during the post‐irradiation period (*n* =; 4). (C) Representative H&E‐stained images of SMG tissues during the post‐irradiation period (*n* = 3). (D) Representative IHC images showing AQP5 expression in SMG tissues during the post‐irradiation period and (E) corresponding semiquantitative analysis (*n* = 3). Scale bars = 100 µm. Data are presented as mean ± SD. All experiments were independently repeated three times. *p < 0.05; **p< 0.01; ***p < 0.001.
**Supplementary Fig. S2**: Immunofluorescence images of α‐SMA and TUNEL assay in SMG. (A) Representative immunofluorescence images showing α‐SMA expression in SMG at different post irradiation periods and (C) corresponding semiquantitative analysis (*n* = 3). (B) Representative TUNEL‐positive cells in SMG at different post irradiation periods and (D) corresponding semiquantitative analysis (*n* = 3). Scale bars = 100 µm. Data are presented as mean ± SD. All experiments were independently repeated three times. **p < 0.01; ***p < 0.001.
**Supplementary Fig S3**: Different expression proteins at different post irradiation days. (A) Differentially expressed proteins and enriched pathways between day 0 and day 1. (B) Differentially expressed proteins and enriched pathways between day 1 and day 4. (C) Differentially expressed proteins and enriched pathways between day 4 and day 8. (D) Differentially expressed proteins and enriched pathways between day 8 and day 22.
**Supplementary Fig S4**: Metabolomic profiling of spermidine dynamics in SMG during radiation‐induced injury. Relative spermidine abundance in SMG tissue was extracted from untargeted metabolomic data at 0, 1, 4, 8, and 22 days after irradiation.
**Supplementary Fig S5**: Design of study and patients enrolled process.
**Supplementary Fig S6**: Quality of life in patients undergoing oral placebo or spermidine supplementation. (A) Physical function. (B) Role functioning. (C) Emotional function. (D) Global health status. (E) Fatigue. (F) Pain. (G) Insomnia. (H) Appetite loss. (I) HN‐swallowing. (J) HN‐senses problems. (K) HN‐opening mouth. (L) HN‐dry mouth. Data are presented as mean ± SD. ns = not statistically significant; *p < 0.05; ** *p* < 0.01; **** *p* < 0.0001. *p* values are adjusted via Benjamini‐Hochberg method.
**Supplementary Fig S7**: Survival patterns in patients undergoing oral placebo or spermidine supplementation. (A) Disease free survival. (B) Overall survival.
**Table S1**: Incidence of xerostomia in two groups.
**Table S2** Summary of adverse events in all patients.
**Table S3**: List of reagents, suppliers and catalog number used in this study.
**Table S4**: List of suppliers, host species, dilution, and clone information of antibodies used in this study.

## Data Availability

The datasets generated and/or analyzed during the current study are available from the corresponding author on reasonable request.
