# Supplementary material for: Oral Spermidine Supplementation Preserves Submandibular Gland Function After Radiotherapy: Mechanistic Insights and Use in a Phase II Randomized Clinical Trial
Source: MedComm (2020). 2026 Jul 15;7(8):e70862. doi: 10.1002/mco2.70862 (PMC13370102; doi:10.1002/mco2.70862)
Supplement: Supplementary file 1 — Supplementary Fig. S1: Construction of the SMG injury model in vivo. (A) Irradiation area for construction the SMG injury model in mice. (B) Salivary flow rates during the post‐irradiation period (n =; 4). (C) Representative H&E‐stained images of SMG tissues during the post‐irradiation period (n = 3). (D) Representative IHC images showing AQP5 expression in SMG tissues during the post‐irradiation period and (E) corresponding semiquantitative analysis (n = 3). Scale bars = 100 µm. Data are presented as mean ± SD. All experiments were independently repeated three times. *p < 0.05; **p< 0.01; ***p < 0.001. Supplementary Fig. S2: Immunofluorescence images of α‐SMA and TUNEL assay in SMG. (A) Representative immunofluorescence images showing α‐SMA expression in SMG at different post irradiation periods and (C) corresponding semiquantitative analysis (n = 3). (B) Representative TUNEL‐positive cells in SMG at different post irradiation periods and (D) corresponding semiquantitative analysis (n = 3). Scale bars = 100 µm. Data are presented as mean ± SD. All experiments were independently repeated three times. **p < 0.01; ***p < 0.001. Supplementary Fig S3: Different expression proteins at different post irradiation days. (A) Differentially expressed proteins and enriched pathways between day 0 and day 1. (B) Differentially expressed proteins and enriched pathways between day 1 and day 4. (C) Differentially expressed proteins and enriched pathways between day 4 and day 8. (D) Differentially expressed proteins and enriched pathways between day 8 and day 22. Supplementary Fig S4: Metabolomic profiling of spermidine dynamics in SMG during radiation‐induced injury. Relative spermidine abundance in SMG tissue was extracted from untargeted metabolomic data at 0, 1, 4, 8, and 22 days after irradiation. Supplementary Fig S5: Design of study and patients enrolled process. Supplementary Fig S6: Quality of life in patients undergoing oral placebo or spermidine supplementation. (A) Physi [file MCO2-7-e70862-s001.docx]

**Supplementary Information**

**Oral Spermidine Supplementation Preserves Submandibular Gland Function After Radiotherapy: Mechanistic Insights and Use in a Phase II Randomized Clinical Trial**

Yu Min ^1, 2#^, Kun Gao ^3#^, Yingtong Liu ^4#^, Lei Dai ^4*^, Xingchen Peng ^1, 2*^

1. Department of Biotherapy, Cancer Center, West China Hospital, Sichuan University, Chengdu, P.R. China.

2. Sichuan Provincial Key Laboratory of Nuclear Physics and Medical Research, Sichuan University, Chengdu, Sichuan, P.R. China.

3. Department of Head and Neck Oncology, Cancer Center, West China Hospital, Sichuan University, Chengdu, P.R. China.

4. Department of Biotherapy, Cancer Center and State Key Laboratory of Biotherapy, West China Hospital, Sichuan University, Chengdu, P.R. China.

^#^ Yu Min, Kun Gao, and Yingtong Liu contributed equally to this work.

^*^Corresponding authors: Xingchen Peng, E-mail: [pxx2014@163.com](mailto:pxx2014@163.com); Lei Dai, E-mail: [daileisklb2012@163.com](mailto:daileisklb2012@163.com) or [dailei2016@scu.edu.cn](mailto:daileisklb2012@163.com)

**Contents**

**Supplementary Figures**: S1-S7

**Supplementary Tables**: S1-S4


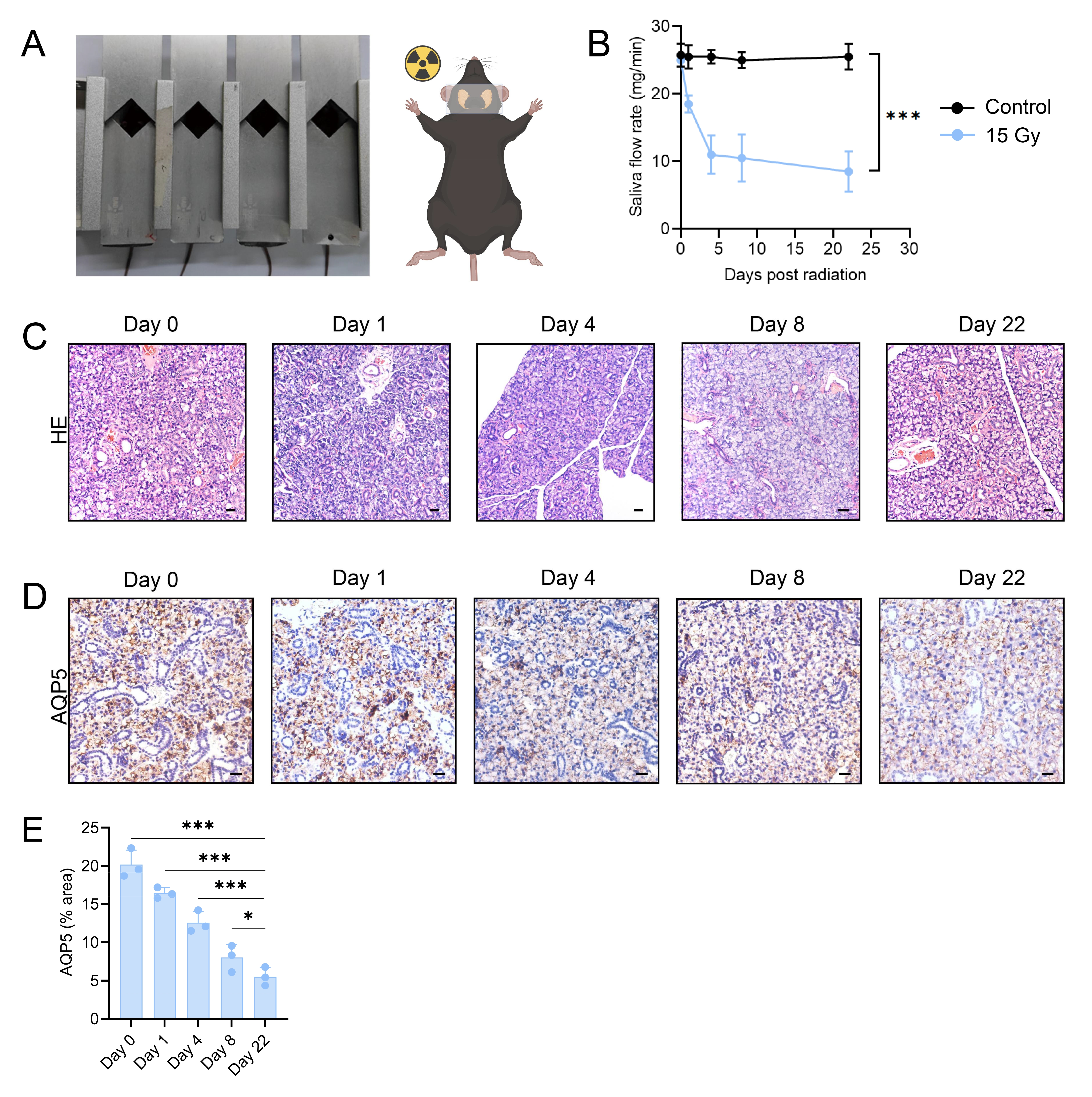


**Supplementary Fig. S1. Construction of the SMG injury model *in vivo*.** (A) Irradiation area for construction the SMG injury model in mice. (B) Salivary flow rates during the post-irradiation period (n = 4). (C) Representative H&E-stained images of SMG tissues during the post-irradiation period (n = 3). (D) Representative IHC images showing AQP5 expression in SMG tissues during the post-irradiation period and (E) corresponding semiquantitative analysis (n = 3). Scale bars = 100 μm. Data are presented as mean ± SD. All experiments were independently repeated three times. **P*<0.05; ***P*<0.01; ****P* < 0.001.


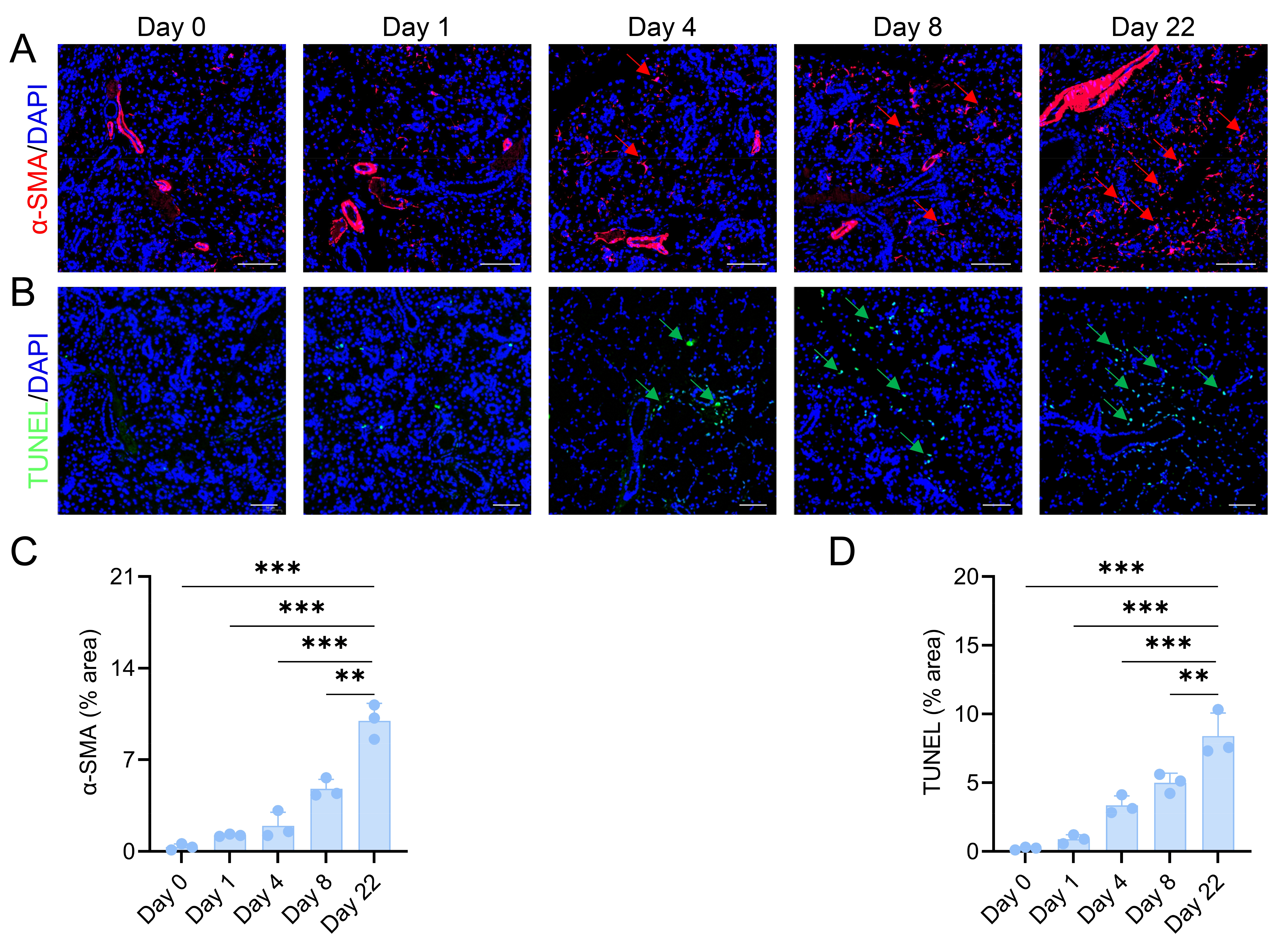


**Supplementary Fig. S2. Immunofluorescence images of α-SMA and TUNEL assay in SMG.** (A) Representative immunofluorescence images showing α-SMA expression in SMG at different post irradiation periods and (C) corresponding semiquantitative analysis (n = 3). (B) Representative TUNEL-positive cells in SMG at different post irradiation periods and (D) corresponding semiquantitative analysis (n = 3). Scale bars = 100 μm. Data are presented as mean ± SD. All experiments were independently repeated three times. ***P*<0.01; ****P* < 0.001.


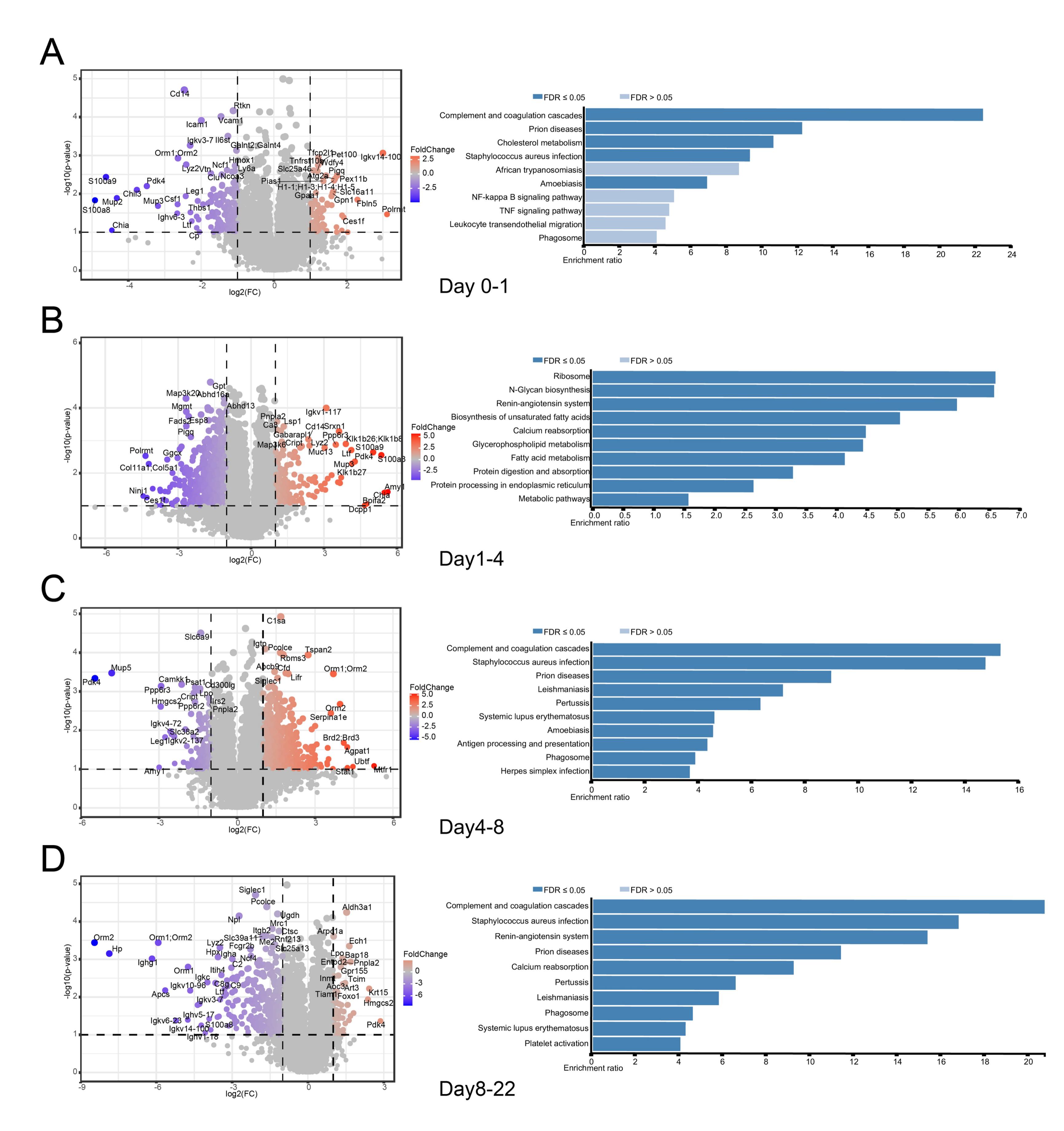


**Supplementary Fig S3. Different expression proteins at different post irradiation days.** (A) Differentially expressed proteins and enriched pathways between day 0 and day 1. (B) Differentially expressed proteins and enriched pathways between day 1 and day 4. (C) Differentially expressed proteins and enriched pathways between day 4 and day 8. (D) Differentially expressed proteins and enriched pathways between day 8 and day 22.


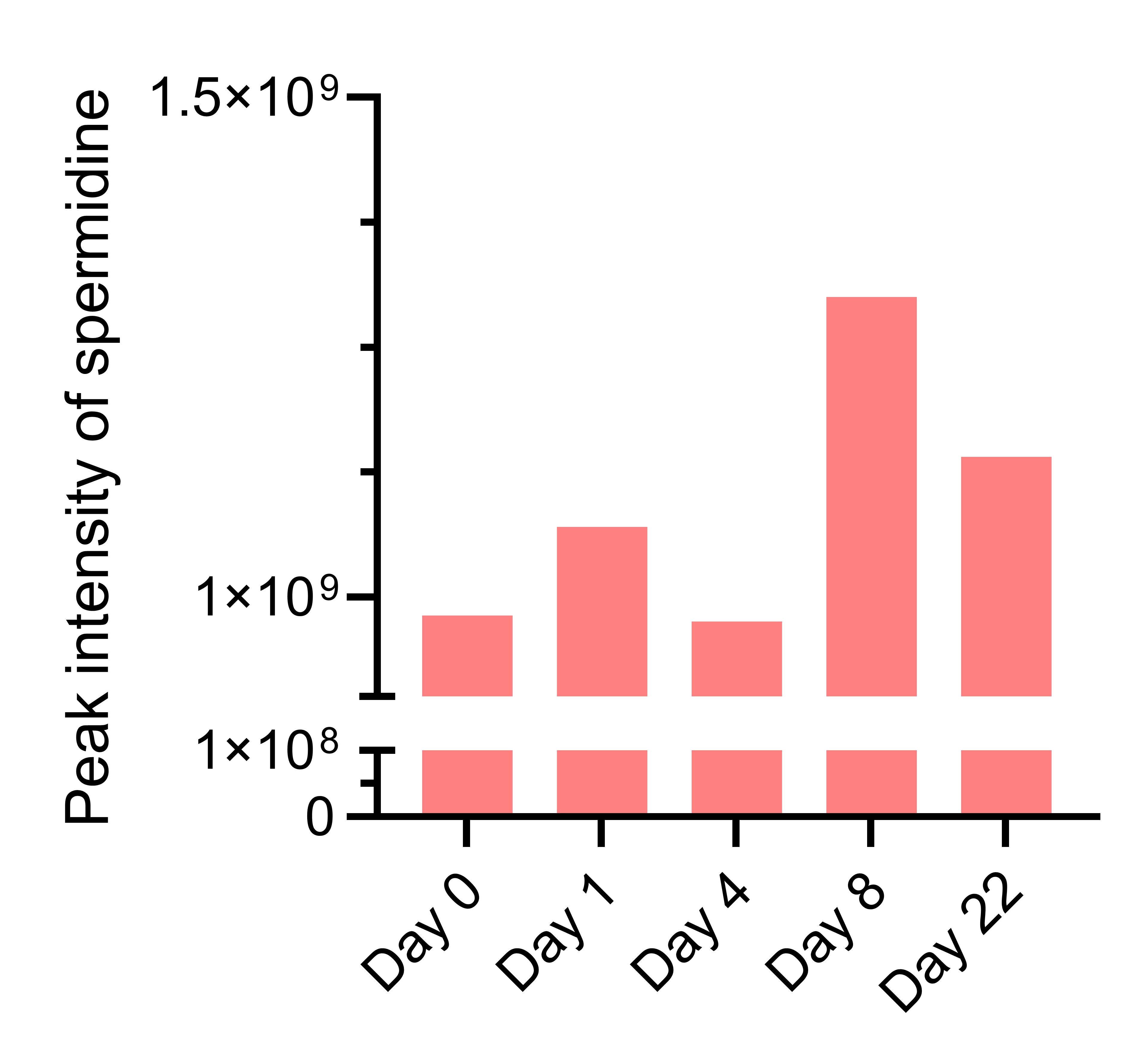


**Supplementary Fig S4.** **Metabolomic profiling of spermidine dynamics in SMG during radiation-induced injury.** Relative spermidine abundance in SMG tissue was extracted from untargeted metabolomic data at 0, 1, 4, 8, and 22 days after irradiation.


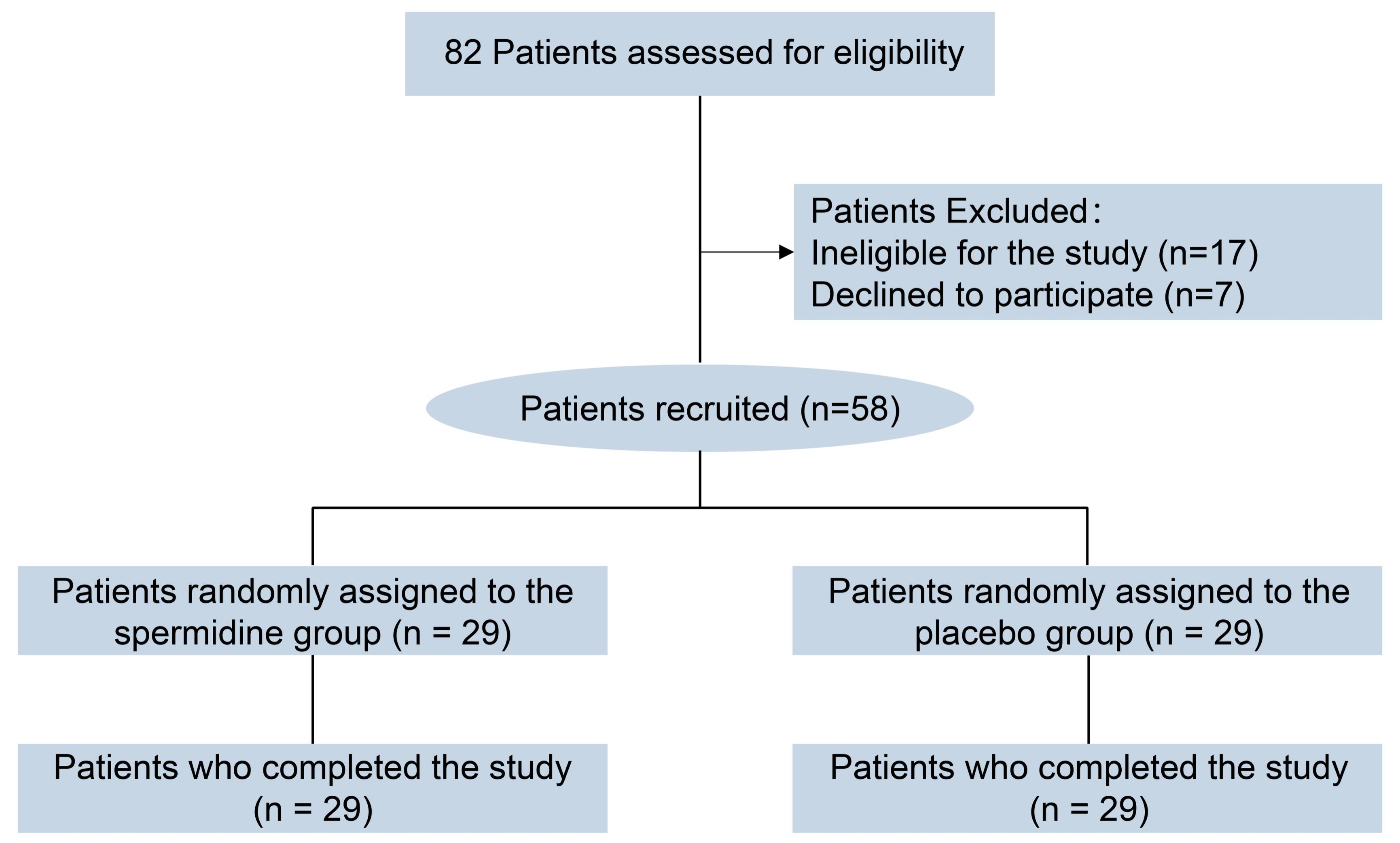


**Supplementary Fig S5. Design of study and patients enrolled process.**


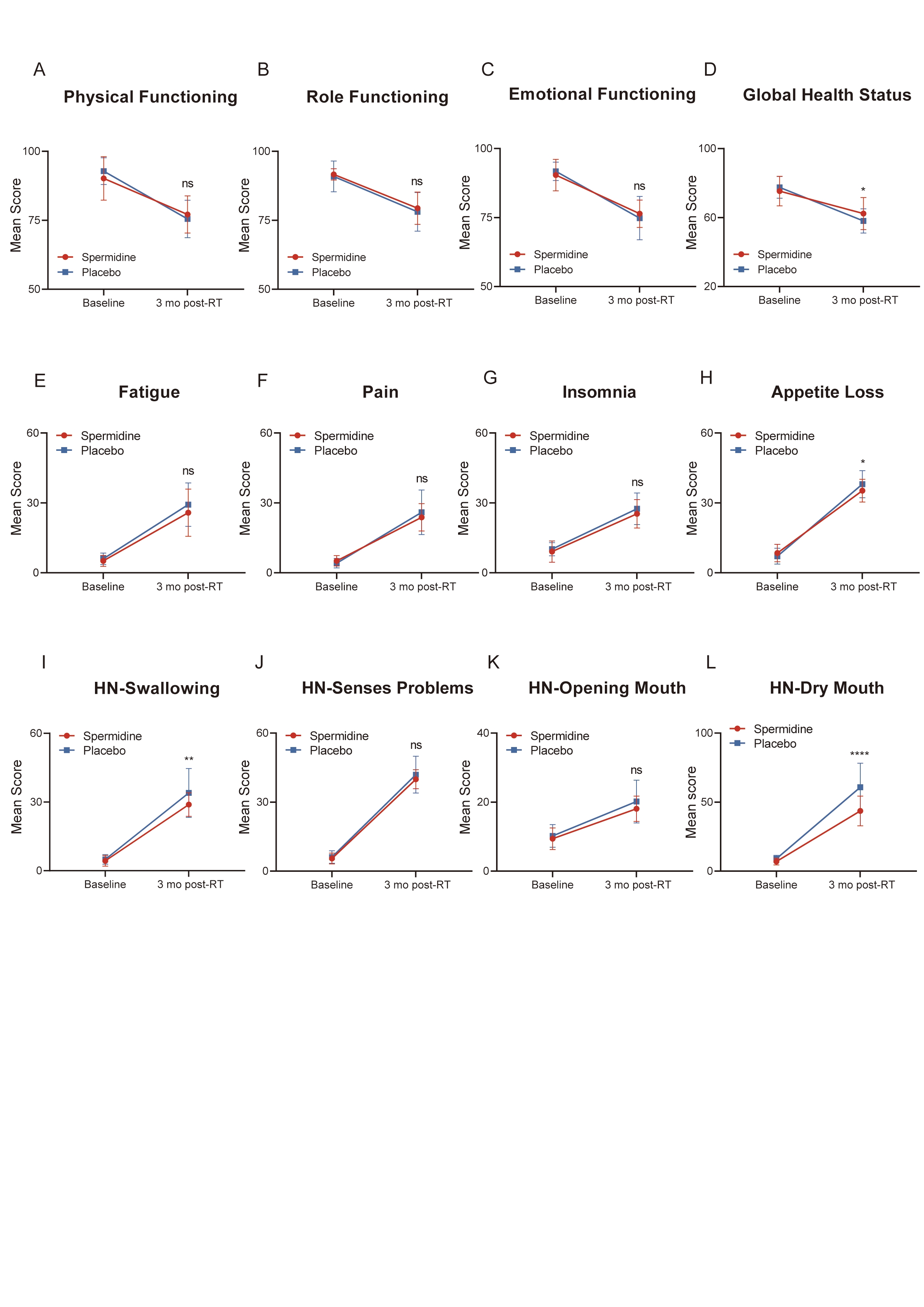


**Supplementary Fig S6. Quality of life in patients undergoing oral placebo or spermidine supplementation.** (A) Physical function. (B) Role functioning. (C) Emotional function. (D) Global health status. (E) Fatigue. (F) Pain. (G) Insomnia. (H) Appetite loss. (I) HN-swallowing. (J) HN-senses problems. (K) HN-opening mouth. (L) HN-dry mouth. Data are presented as mean ± SD. ns = not statistically significant; ^*^ *P* < 0.05; ^**^ *P* < 0.01; ^****^ *P* < 0.0001. *P* values are adjusted via Benjamini-Hochberg method.


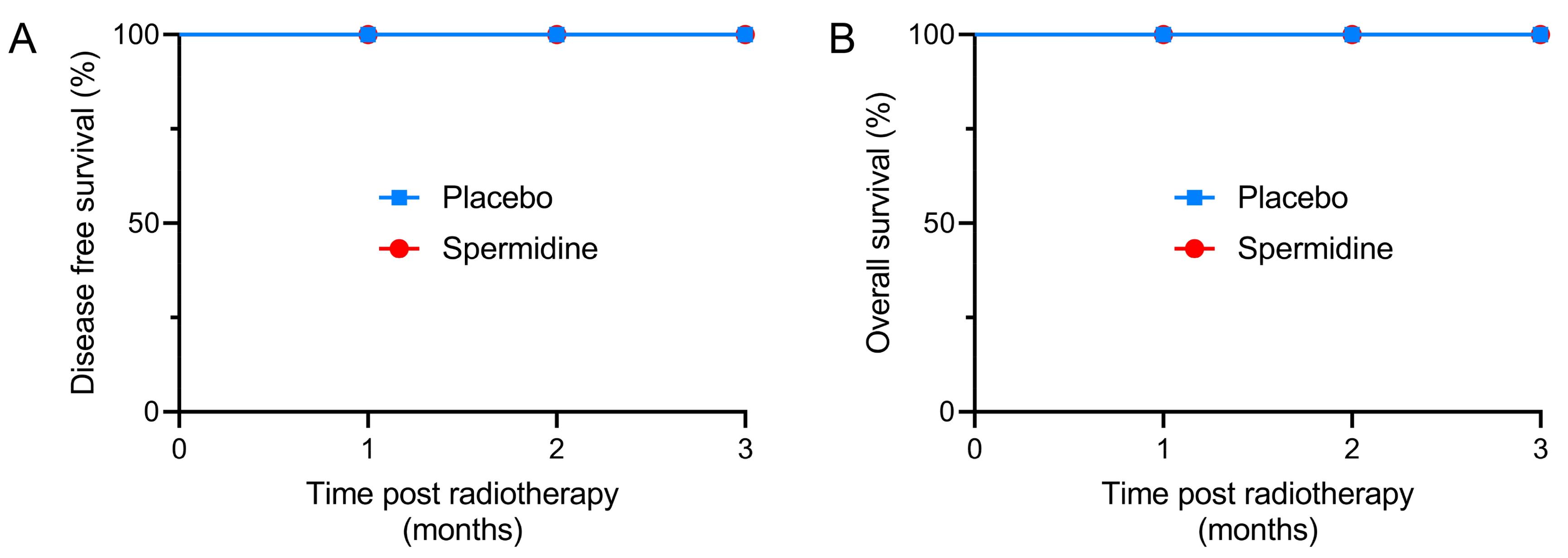


**Supplementary Fig S7. Survival patterns in patients undergoing oral placebo or spermidine supplementation.** (A) Disease free survival. (B) Overall survival.

**Table S1.** Incidence of xerostomia in two groups.

| Time | Incidence of Placebo Group (%) | Incidence of Spermidine Group (%) | Rate Differences | Standard Errors | One-Sided 95% CI Upper Limits | *Z* Values | *P* |
| --- | --- | --- | --- | --- | --- | --- | --- |
| Baseline | 0 | 0 | 0 | 0 | 0 | NA | 1 |
| Mid-RT | 20.69% | 17.24% | -3.45% | 0.1029 | 0.1347 | 0.6370 | 0.7379 |
| End-RT | 51.72% | 48.28% | -3.44% | 0.1312 | 0.1814 | 0.4993 | 0.6912 |
| 1 mo post-RT | 86.21% | 75.86% | -10.35% | 0.1021 | 0.0644 | -0.0338 | 0.4865 |
| 2 mo post-RT | 82.76% | 68.97% | -13.79% | 0.1109 | 0.0445 | -0.3420 | 0.3662 |
| 3 mo post-RT | 79.31% | 48.28% | -31.03% | 0.1195 | -0.1139 | -1.7609 | **0.0391** |

Abbreviations: NA, not applicable; 1 mo post-RT, 1 month after the conclusion of radiotherapy; 2 mo post-RT, 2 months after the conclusion of radiotherapy; 3 mo post-RT, 3 months after the conclusion of radiotherapy.

**Table S2. Summary of adverse events in all patients.**

| **Adverse events** | **No. (%) of patients** | |
| --- | --- | --- |
|  | **Spermidine**  **(N = 29)** | **Placebo**  **(N = 29)** |
| Abdominal distension | 2 (6.9) | 1 (3.4) |
| Diarrhea | 1 (3.4) | 0 (0.0) |

Abbreviations: N: number.

**Table S3.** List of reagents, suppliers and catalog number used in this study.

| Reagents | Suppliers | Catalog number |
| --- | --- | --- |
| 10% Bovine Serum Albumin (BSA) solution | Gibco | A5256501 |
| Hanks’ Balanced Salt Solution | Gibco | 14170112 |
| Red Blood Cell Lysis Buffer | Solarbio | [R1010](https://www.solarbio.com/goodsInfo?id=450) |
| Collagenase II | Thermo Fisher Scientific | 17101015 |
| Hyaluronidase | Merck | HX0514 |
| Calcium Chloride | Merck | 10043-52-4 |
| 70 µm cell strainer | Corning | 431751 |
| Trypsin | Gibco | 25200072 |
| Matrigel® | Corning | [356234](https://ecatalog.corning.com/life-sciences/b2c/US/en/Surfaces/Extracellular-Matrices-ECMs/Corning%C2%AE-Matrigel%C2%AE-Matrix/p/356234) |
| Dulbecco’s Modified Eagle Medium | Gibco | 11965092 |
| 1% Penicillin / Streptomycin | Gibco | 15140-122 |
| GlutaMAX Supplement | Thermo Fisher Scientific | 35050-061 |
| Epidermal Growth Factor | PeproTech | AF‑100‑15 |
| Fibroblast Growth Factor 2 | PeproTech | 100‑18B |
| Hepatocyte Growth Factor (HGF) | Yeasen | 92055ES |
| N-2 Supplement | Gibco | 17502-048 |
| Dexamethasone | Merck | 50-02-2 |
| Insulin | Merck | EZRMI |
| γ‑Secretase Inhibitor | Merck | 565784-M |
| MobiCub® 3’ Transcriptome Bead Kit 2.0 | Zhejiang Mozhuo Bio‑tech | PN‑S050400201 |
| MobiCube® 3’Transcriptome Reverse Transcription Kit 2.0 | Zhejiang Mozhuo Bio‑tech | PN‑S050500201 |
| Agilent High Sensitivity DNA Kit | Agilent Technologies | PN‑S050600201 |
| Golgi‑Tracker Red | Beyotime | C1043 |
| Cell‑Light EdU Apollo 567 In Vitro Kit | Ruibo | C10310-1 |
| One‑Step TUNEL Apoptosis Detection Kit | Beyotime | FD014 |
| Hematoxylin and Eosin Staining Kit | Beyotime | C0105S |
| Nissl Staining Kit | Beyotime | C0117 |
| EDTA Antigen Retrieval Buffer | Zhongshan Golden Bridge | ZLI-9069 |
| DAPI Anti‑Fade Mounting Medium | Beyotime | P0131 |
| PBS Buffer Powder | Beyotime | ST447 |
| SpermidineLIFE® | **Longevity Labs+** | - |
| Spermidine | **Sigma** | 85558 |

**Table S4.** List of suppliers, host species, dilution, and clone information of antibodies used in this study.

| **Antibodies** | **Suppliers** | **Catalog No.** | **Host species** | **Clone** | **Dilution** |
| --- | --- | --- | --- | --- | --- |
| AQP5 antibody | Abcam | ab315855 | Rabbit | Polyclonal | 1:1000 |
| α-Smooth Muscle Actin (α‑SMA) antibody | Proteintech | 14395-1-AP | Rabbit | Polyclonal | 1:1500 |
| Goat anti‑rabbit IgG | Proteintech | SA00001-2 | Goat | Polyclonal | 1:1000 |
| Goat anti‑mouse IgG | Proteintech | SA00001-1 | Goat | Polyclonal | 1:1000 |
